# A Review of AI Cloud and Edge Sensors, Methods, and Applications for the Recognition of Emotional, Affective and Physiological States

**DOI:** 10.3390/s22207824

**Published:** 2022-10-14

**Authors:** Arturas Kaklauskas, Ajith Abraham, Ieva Ubarte, Romualdas Kliukas, Vaida Luksaite, Arune Binkyte-Veliene, Ingrida Vetloviene, Loreta Kaklauskiene

**Affiliations:** 1 Department of Construction Management and Real Estate, Vilnius Gediminas Technical University, Sauletekio Ave. 11, LT-10223 Vilnius, Lithuania; 2 Machine Intelligence Research Labs, Scientific Network for Innovation and Research Excellence, Auburn, WA 98071, USA; 3 Institute of Sustainable Construction, Vilnius Gediminas Technical University, Sauletekio Ave. 11, LT-10223 Vilnius, Lithuania; 4 Department of Applied Mechanics, Vilnius Gediminas Technical University, Sauletekio Ave. 11, LT-10223 Vilnius, Lithuania

**Keywords:** review, human emotions, affective and physiological states, Plutchik’s wheel of emotions, sensors, methods and applications, statistical and multiple criteria analysis, country success and publications maps of the world

## Abstract

Affective, emotional, and physiological states (AFFECT) detection and recognition by capturing human signals is a fast-growing area, which has been applied across numerous domains. The research aim is to review publications on how techniques that use brain and biometric sensors can be used for AFFECT recognition, consolidate the findings, provide a rationale for the current methods, compare the effectiveness of existing methods, and quantify how likely they are to address the issues/challenges in the field. In efforts to achieve the key goals of Society 5.0, Industry 5.0, and human-centered design better, the recognition of emotional, affective, and physiological states is progressively becoming an important matter and offers tremendous growth of knowledge and progress in these and other related fields. In this research, a review of AFFECT recognition brain and biometric sensors, methods, and applications was performed, based on Plutchik’s wheel of emotions. Due to the immense variety of existing sensors and sensing systems, this study aimed to provide an analysis of the available sensors that can be used to define human AFFECT, and to classify them based on the type of sensing area and their efficiency in real implementations. Based on statistical and multiple criteria analysis across 169 nations, our outcomes introduce a connection between a nation’s success, its number of Web of Science articles published, and its frequency of citation on AFFECT recognition. The principal conclusions present how this research contributes to the big picture in the field under analysis and explore forthcoming study trends.

## 1. Introduction

Global research in the field of neuroscience and biometrics is shifting toward the widespread adoption of technology for the detection, processing, recognition, interpretation and imitation of human emotions and affective attitudes. Due to their ability to capture and analyze a wide range of human gestures, affective attitudes, emotions and physiological changes, these innovative research models could play a vital role in areas such as Industry 5.0, Society 5.0, the Internet of Things (IoT), and affective computing, among others.

For hundreds of years, researchers have been interested in human emotions. Reviews on the applications of affective neuroscience include numerous related topics, such as the mirror mechanism and its role in action and emotion [1], the neuroscience of under-standing emotions [2], consumer neuroscience [3], the role of positive emotions in education [4], mapping the brain as the basis of feelings and emotions [5], the neuroscience of positive emotions and affect [6], the cognitive neuroscience of music perception [7], and social cognition in schizophrenia [8]. Applications in neuroscience also include the analysis of cognitive neuroscience [9,10,11], and brain sensors [12,13], and works in the literature also discuss the recognition of basic emotions using brain sensors [14].

Studies of the applications of affective biometrics can be found in the literature in the fields of brain biometric analysis [15], predictive biometrics [16], keystroke dynamics [17], applications in education [18], consumer neuroscience [19], adaptive biometric systems [20], emotion recognition from gait analyses [21], ECG databases [22], and others. Several works on affective states have integrated multiple biometric and neuroscience methods, but none have included an integrated review of the application of neuroscience and biometrics and an analysis of all of the emotions and affective attitudes in Plutchik’s wheel of emotions.

Scientists analyzed various brain and biometric sensors in the reviews [23,24,25,26]. Curtin et al. [23], for instance, state that both fNIRS and rTMS sensors have changed significantly over the past decade and have been improved (their hardware, neuronavigated targeting, sensors, and signal processing), thus clinicians and researchers now have more granular control over the stimulation systems they use. Krugliak and Clarke [26], da Silva [24], and Gramann et al. [27] analyzed the use of EEG and MEG sensors to measure functional and effective connectivity in the brain. Khushaba et al. [25] used brain and biometric sensors to integrate EEG and eye tracking for assessing the brain response. Other scientists [28,29,30,31,32,33] used the following biometric sensors in their studies: heart rate, pulse rate variability, odor, pupil dilation and contraction, skin temperature, face recognition, voice, signature, gestures, and others.

Indeed, the biometrics and neuroscience field has been the focus of studies by many researchers who have achieved significant results. A number of neuroscience studies have analyzed the detection and recognition of human arousal [34], valence [35,36], affective attitudes [36,37], emotional [38,39,40,41], and physiological [42] states (AFFECT) by capturing human signals.

Though most neuroimaging approaches disregard context, the hypothesis behind situated models of emotion is that emotions are honed for the current context [43]. According to the theory of constructed emotion, the construction of emotions should be holistic, as a complete phenomenon of brain and body in the context of the moment [44]. Barrett [45] argues that rather than being universal, emotions differ across cultures. Emotions are not triggered—they are created by the person who experiences them. The combination of the body’s physical characteristics, the brain (which is flexible enough to adapt to whatever environment it is in), and the culture and upbringing that create that environment, is what causes emotions to surface [45]. Recently, there have been attempts in the academic community to supply contextual (from cultural and other circumstances) analysis [46,47].

Various theories and approaches (positive psychology [48,49,50], environmental psychology [51,52,53], ergonomics—human factors science [54,55,56], environment–behavior studies, environmental design [57,58,59], ecological psychology [60,61], person–environment–behavior [62], behavioral geography [63], and social ecology research [64] also emphasize emotion context sensitivity.

The objective of this research is to provide an overview of the sensors and methods used in AFFECT (affective, emotional, and physiological states) recognition, in order to outline studies that discuss trends in brain and biometric sensors, and give an integrated review of AFFECT recognition analysis using Plutchik’s [65] wheel of emotions as the basis. Furthermore, the research aim is to review publications on how techniques that use brain and biometric sensors can be used for AFFECT recognition. In addition, this is a quantitative study to assess how the success of the 169 countries impacted the number of Web of Science articles on AFFECT recognition techniques that use brain and biometric sensors that were published in 2020 (or the latest figures available).

In this paper, we identify the critical changes in this field over the past 32 years by applying text analytics to 21,397 articles indexed by Web of Science from 1990 to 2022. For this review, we examined 634 publications in detail. We have analyzed the global gap in the area of neuroscience and affective biometric sensors and have aimed to update the current big picture. The aforementioned research findings are the result of this work. 

When emotions as well as affective and physiological states are determined by recognition sensors and methods—and, later, when such studies are put to practice—a number of issues arise, and we have addressed these issues in this review. Moreover, our research has filled several research gaps and contributes to the big picture as outlined below:

- A fairly large number of studies around the world apply biometric and neuroscience methods to determine and analyze AFFECT. However, there has been no integrated review of these studies.
- Another missing piece is a review of AFFECT recognition, classification, and analysis based on Plutchik’s wheel of emotions theory. We have examined 30 emotions and affective states defined in the theory.
- Information on diversity attitudes, socioeconomic status, demographic and cultural background, and context is missing from many studies. We have therefore identified real-time context data and integrated them with AFFECT data. The correct assessment of AFFECT and predictions of imminent behavior are becoming very important in a highly competitive market.
- To demonstrate a few of the aforementioned new research areas in practice, we have developed our own metric, the Real-time Vilnius Happiness Index (Section 4), among other tools. These studies have used integrated methods of biometrics and neuroscience, which are widely applied in various fields of human activity.
- In this research, we therefore examine a more complex problem than any prior studies.

The following sections present the results of this study, a discussion, the conclusions we can draw, and avenues for future research. The method is presented in Section 2. Section 3 summarizes the emotion models. In Section 4, we discuss about brain and biometrics AFFECT sensors, classifications of biometric and neuroscience methods and technologies, emotions and explores the use of traditional, non-invasive neuroscience methods (Section 4) and widely used and advanced physiological and behavioral biometrics (Section 4). Section 4 also summarizes prior research and studies techniques for the recognition of arousal, valence, affective attitudes, and emotion-al and physiological states (AFFECT) in more detail. We summarize existing research on users’ demographic and cultural backgrounds, socioeconomic status, diversity attitudes, and the context in Section 5. We present our research results in Section 6, evaluation of biometric systems in Section 7, and finally, a discussion and our conclusions in Section 8.

## 2. Method

The research method we used can be broken down as follows: (1) formulating the research problem; (2) examining the most popular emotion models, identifying the best option among them for our research (Section 3), and creating the Big Picture for the model; (3) carrying out a review of publications in the field (Section 4); (4) raising and confirming two hypotheses; (5) collecting data; (6) using the INVAR method for multiple criteria analysis of 169 countries; (7) determining correlations; (8) developing three maps to illustrate the way the success of the 169 countries impacts the number of Web of Science articles on AFFECT (emotional, affective, and physiological states) recognition and their citation rates; (9) developing three regression models; and (10) consolidating the findings, providing a rationale for the current methods, comparing the effectiveness of existing methods, and quantifying how likely they are to address the issues and challenges in the field. The following ten steps of the method describe the proposed algorithm and its experimental evaluation in detail.

Furthermore, the research aim is to review publications on how techniques that use brain and biometric sensors can be used for AFFECT recognition, consolidate the findings, provide a rationale for the current methods, compare the effectiveness of existing methods, and quantify how likely they are to address the issues/challenges in the field (Step 1). We have analyzed the global gap in the area of neuroscience and affective biometric sensors and have set the goal of updating the current big picture. The findings of the research above framed the problem.

Step 2 of the research was to examine the most popular emotion models (Section 3) and identify the best option among them for our research. We have chosen the Plutchik’s wheel of emotions and one of the main reasons is that the model enables integrated analysis of human emotional, affective, and physiological states. 

Step 3 was to review sensors, methods, and applications that can be used in the recognition of emotional, affective, and physiological states (Section 4). We have identified the major changes in the field over the past 32 years through a text analysis of 21,397 articles indexed by Web of Science from 1990 to 2022. We searched for keywords in three databases (Web of Science, ScienceDirect, Google Scholar) to identify studies investigating the use of both neuroscience and affective biometric sensors. A total of 634 studies that used both neuroscience and affective biometric sensor techniques in the study methodology were included, and no restrictions were placed on the date of publication. Studies which investigated any population group were at any age or gender were considered in this work.

A set of keywords related to biometric and neuroscience sensors were used for the above search of three databases. Two main sets of keywords “sensors + biometrics + emotions” and “sensors + neuroscience/brain + emotions” were used in our main search. More specific search terms related to biometrics (i.e., eye tracking, blinking, iris, odor, heart rate), neuroscience/brain techniques (i.e., EEG, MEG, TMS, NIRS, SST) and their components (i.e., algorithms, functionality, performance) were also used to refine the search. For each candidate article, the full text was accessed and reviewed to determine its eligibility. The primary results and article conclusions were identified, and discrepancies were resolved by way of discussion. The studies differed significantly in terms of protocol design, signal processing, stimulation methods, the equipment used, the study population, and statistical methods.

In Step 4, two central hypotheses were raised and confirmed:

**Hypothesis** **1.** *There is an interconnection between a country’s success, its number of Web of Science articles published, and its citation frequency on AFFECT recognition. When there are changes in the country’s success, its number of Web of Science articles published, and its citation times on AFFECT recognition, the countries’ 7 cluster boundaries remain roughly the same (* Section 6 *).*

**Hypothesis** **2.** *Increases in a country’s success usually go hand in hand with a jump in its number of Web of Science articles published and its citation times on AF-FECT recognition.*

Next, in Step 5, we collected data. The determination of the success of 169 countries and the results obtained are described in detail in a study by Kaklauskas et al. [66]. This study used data [66] from the framework of variables taken from a number of databases and websites, such as the World Bank, Eurostat-OECD, the World Health Organization, Global Data, Global Finance, Transparency International, Freedom House, Knoema, Socioeconomic Data and Applications Center, Heritage, the Global Footprint Network, Climate Change Knowledge Portal (World Bank Group, Washington, DC, USA), the Institute for Economics and Peace, and Our World in Data; global and national statistics and publications were also used. We based our research calculations on publicly available data from 2020 (or the latest available).

We used the INVAR method [67] to conduct a multi-criteria examination of the 169 nations—the outcomes can be found in Section 6 (Step 6). This method determines a combined indicator for whole nation success. This combined indicator is in direct proportion to the corresponding impact of the values and significances of the specified indicators on a nation’s success. The INVAR method was used to conduct multiple criteria analyzes of different groups of countries, such as the former Soviet Union [68], Asian countries [69], and the global analysis of 169 [66] and 173 [70] countries.

The study’s 7th step presents the median values of the correlations for 169 countries, its publications, and citations (Section 6). It was found that the median correlation of the dependent variable of the Publications—Country Success model with the independent variables (0.6626) is higher than in the Times Cited—Country Success model (0.5331). Therefore, it can be concluded that the independent variables in the Publications—Country Success model are more closely related to the dependent variable than in the Times Cited—Country Success model.

In Step 8, we developed three maps that illustrate the way the success of the 169 countries impacts the number of Web of Science articles on AFFECT (emotional, affective, and physiological states) recognition and their citation rates. The Country’s Success and AFFECT Recognition Publications (CSP) Maps of the World are a convenient way to illustrate how the three predominant CSP dimensions (a country’s success, the numbers of publications, and the frequency of articles being cited) are interconnected for the 169 countries, while the CSP models allow for these connections to be statistically analyzed from various perspectives. It also allows for CSP dimensions to be forecast based on the country’s success criteria. In other words, the CSP models give us a more detailed analysis of the CSP dimensions through statistical and multi-criteria analysis, while the CSP maps (Section 6) are more of a way to present the results in a visual manner. The amount of data available is gradually increasing, as is the knowledge gained from research conducted around the world. As a result, the CSP models are becoming better and better, and providing a clearer reflection of the actual picture. This means that they can effectively facilitate research and innovation policy decisions. 

In Step 9, we created two regression models (Section 6). For the multiple linear regressions, we used IBM SPSS V.26 to build two regression models on 15 indicators of country success [66] and the three predominant CSP dimensions (Section 6). Step 9 entailed the construction of regression models for the number of publications and their citation rates, and the calculation of the effect size indicators describing them. Two dependent variables and 15 independent variables were analyzed to construct these regression models. The process was as follows:

- Construction of regression models for the numbers of publications and their citations.
- Calculation of statistical (Pearson correlation coefficient (r), standardized beta coefficient (β), coefficient of determination (R^2^), standard deviation, *p*-values) and non-statistical (research context, practical benefit, indicators with low values) effect size indicators describing these regression models.

It was found that changes in the values of the Country Success variable explain the variance of the Publications variable by 89.5%, and the variance of the Times Cited variable by 54.0%. Additionally, when the value of the Country Success variable increases by 1%, the value of Publications increases by 1.962% and Times Cited—by 2.101%. As the success of a country increased by 1%, the numbers of Web of Science articles published and their citations grew by 1.962% and 2.101%, respectively. A reliability analysis of the compiled regression models allows us to conclude that the models are suitable for analysis (*p* < 0.05). The 15 country success indicators explained 69.4% and 51.18% of the number of Web of Science articles published and their citations, respectively.

Step 10 was to assess the biometric systems under analysis: the rationale behind the available biometric and brain approaches was outlined, the efficacy of existing methods compared, and their ability to address issues and challenges present in the field determined (Section 7).

## 3. Emotion Models

First, this chapter will discuss emotion models in more detail. Then, we will choose the best option for our research and look at the Big Picture, i.e., the links between the selected emotion model and biometric and brain sensors, and the trends. 

Emotional responses are natural to humans, and evidence shows they influence thoughts, behavior, and actions. Emotions fall into different groups related to various affects, corresponding to the current situation that is being experienced [71]. People encounter complex interactions in real life, and respond to them with complex emotions that often can be blends [72]. Emotional responses are the way for our brain and body to deal with our environment, and that is why they are fluid and depend on the context around us [73].

Two fundamental viewpoints form the basis in approaches to the classification of emotions: (a) emotions are discrete constructs and they have fundamental differences, and (b) emotions can be grouped and characterized on a dimensional basis [74]. These classifications (emotions as discrete categories and dimensional models of emotion) are briefly analyzed next.

In word recognition, alternative models have so far received little interest, and one example is the discrete emotion theory [75]. This theory posits that there is a limited set of universal basic emotions hardwired through evolution, and that each of the wide variety of affective experiences can essentially be categorized into this limited set [76,77]. The discrete emotion theory states that many emotions can be distinguished on the basis of expressive, behavioral, physiological, and neural features [78]. The definition of emotions provided by Fox [79] states they are consistent and discrete responding processes that can include verbal, physiological, behavioral, and neural mechanisms. They are triggered and changed by external or internal stimuli or events and respond to the environment. Russell and Barrett [80] argue that, unlike the discrete emotion theory, their alternative models can account for the rich context-sensitivity and diversity of emotions. Emotion blends could be of three kinds: (a) Positive-blended emotions were blends of only positive emotions; (b) negative-blended emotions were blends of only negative emotions; and (c) mixed emotions were blends of both positive and negative emotions, as well as neutral ones. The way teachers have described blended emotions reflects that mathematics teaching involves many and complex tasks, where the teacher has to continuously keep gauging the level of progress [81].

Emotional dimensions represent the classes of emotion. Categorized emotions can be characterized in a dimensional form, with each emotion located in a different location in space, for example in 2D (the circumplex model, “consensual” model of emotion, and vector model) or 3D (the Lövheim cube, the pleasure–arousal–dominance (PAD) emotional state model, and Plutchik’s model) [82]. 

The circumplex model [83] proposes that two independent neurophysiological systems: One of the systems is related to arousal (activated/deactivated) and to valence (a pleasure–displeasure continuum), and the other to valence (a continuum from pleasure to displeasure) and to arousal (activation–deactivation) [84]. Each emotion can be understood as having varying valence and arousal, and is a linear combination of these two dimensions, or as varying valence and arousal [83,85]. We already applied the Russel’s circumplex model of emotions to perform a review of the human emotion recognition of sensors and methods [85]. 

The vector model comprises two vectors. The model holds that there is an underlying dimension of arousal with a binary choice of valence that determines direction, and an underlying dimension of arousal. This results in there being two vectors that, both starting at zero arousal and neutral valence and zero arousal, proceed as straight lines, one in a positive, and one in the direction of negative valence and the other in the direction of positive valence. Typically, the vector model uses direct scaling of the dimensions of each individual stimulus individually in this model [86,87].

The positive activation–negative activation (PANA) or “consensual” model of emotion, also known as positive activation/negative activation (PANA), assumes that there are two separate systems—positive affect and negative affect. In the PANA model, the vertical axis represents low to high positive affect, and the horizontal axis of this model represents low to high negative affect (low to high). The vertical axis represents positive affect (low to high) [88]. There are two uncorrelated and independent dimensions: Positive Affect (PA), represents the extent (from low to high) to which a person shows enthusiasm for life. The second factor is Negative Affect (NA), and NA represents the extent to which a person is feeling upset or unpleasantly aroused. Positive Affect and Negative Affect are independent and uncorrelated dimensions [89].

The Pleasure–Arousal–Dominance (PAD) Emotional-State Model, offers a general three-dimensional approach to measuring emotions [90]. This 3D model captures emotional response, and includes the three dimensions of pleasure–displeasure (P), arousal–nonarousal (A), and dominance–submissiveness (D) as basic factors of emotional response [91]. The initials PAD stand for pleasure, arousal, and dominance, which span different emotions. For instance, pleasure can be happy/unhappy, hopeful/despairing, satisfied/unsatisfied, pleased/annoyed, content/melancholic, and relaxed/bored. Arousal can be excited/calm, stimulated/relaxed, wide-awake/sleepy, jittery/dull, frenzied/sluggish, and aroused/unaroused. Dominance can be important/awed, dominant/submissive, influential/influenced, controlling/controlled, in control/cared-for, and autonomous/guided [92]. The neuro-decision and neuro-correlation tables, the inverted U-curve theory, the PAD emotional state model, neuro-decision making, and neuro-correlation tables are used to evaluate the impact of digital twin smart spaces (such as indoor air quality, a level of the lighting intensity and colors, learning materials, images, smells, music, pollution, and others) on users, and track their response dynamics in real time, and to then react to this response [93].

The PAD is composed of three different subscales, reflecting pleasure, arousal, and dominance. These can represent different emotions; for example, the pleasure states include happy (unhappy), pleased (annoyed), satisfied (unsatisfied), contented (melancholic), hopeful (despairing) and relaxed (bored), while the arousal states include stimulated (relaxed), excited (calm), frenzied (sluggish), jittery (dull), wide awake (sleepy) and aroused (unaroused), and the dominance states include controlling (controlled), influential (influenced), in control (cared for), important (awed), dominant (submissive), and autonomous (guided) [92]. The affective space model makes it possible to visualize the distribution of emotions along the two axes of valance (V) and arousal (A). Using this model, different emotions can be identified, such as happiness, calmness, fear, and sadness [94].

Swedish neurophysiologist Lövheim proposed that a cube of emotion is the direct relation between certain specific combinations of the levels of the three signal substances (serotonin, noradrenaline, and dopamine) and eight basic emotions [95]. A three-dimensional model, the Lövheim cube of emotion, was presented where there is a model with each of the signal substances of form represented as the axes of a coordinated system, and each corner of this 3D space holding one of the eight basic emotions is placed in the eight corners. In this model, anger is produced by the combination of high noradrenaline, high dopamine, and low serotonin [96].

The eight main categories of emotions defined by Robert Plutchik in 1980s include two equal groups opposite to each other: half are positive emotions and the other half are negative ones [97]. To visualize eight primary emotion dimensions, which are fear, trust, surprise, anticipation, anger, joy, disgust and sadness, eight sectors have been isolated [98]. The Emotion Wheel shows each of the eight basic emotions highlighted with a recognizable color [99]. When we add another dimension, the Wheel of Emotions becomes a cone with its vertical dimension representing intensity. Moving from the outside towards the wheel’s center emotions intensify and this fact is highlighted by the indicator color. The intensity of emotions is decreasing towards the outer edge and the color, correspondingly, becomes less intense [98,99]. When feelings intensify one feeling can turn into another: annoyance into rage, serenity into ecstasy, interest into vigilance, apprehension into terror, acceptance into admiration, pensiveness into grief, distraction into amazement, and, if left unchecked, boredom can become loathing [98]. Some emotions have no color marking. They are a mix of two primary emotions [98,99]. Joy and anticipation, for instance, combine to become optimism. When anticipation combines with anger it becomes aggressiveness. The combination of trust and fear is submission, joy and trust combine to become love, surprise and fear become awe, the pair of disgust and anger becomes contempt, sadness and disgust combine to become remorse, and surprise and sadness become disapproval [100].

After the analysis of the said emotion models, we have made the decision to choose Plutchik’s wheel of emotions for our research. The ability to analyze human emotional, affective, and physiological states in an integrated manner offered by this model is one of the main reasons of our choice. The wheel is briefly discussed below.

**Figure 1:**
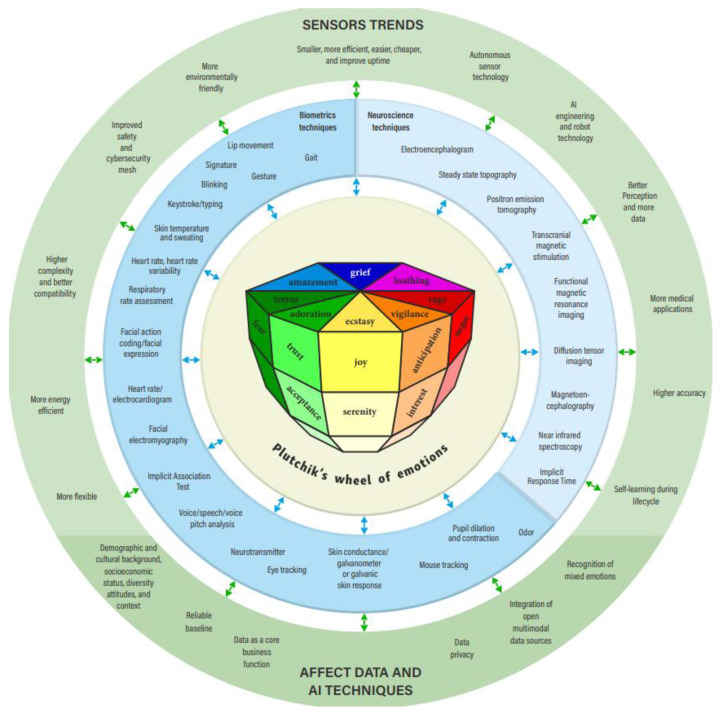
Plutchik’s wheel of emotions, biometrics and neuroscience sensors, and trends.

Several ways to classify emotions have been proposed in the field of psychology. For that purpose, the basic emotions are first identified and then they allow clustering with any other more complex emotion [101]. Plutchik [65] proposed a classification scheme based on eight basic emotions arranged in a wheel of emotions, similar to a color wheel. Just like complementary colors, this setup allows the conceptualization of primary emotions by placing similar emotions next to each other and opposites 180 degree apart. Plutchik’s wheel of emotions classifies these eight basic emotions grounded on the physiological aim [102]. Emotions are coordinated with the body’s physiological responses. For example, when you are scared, your heart rate typically increases and your palms become sweaty. There is ample empirical evidence that suggests that physiological responses accompany emotion [103]. Another parallel with colors is the fact that some emotions are primary emotions and other emotions are derived by combining these primary emotions. The two models share important similarities, and such modelling can also serve as an analytical tool to understand personality. In this case, a third dimension has been added to the circumplex model to represent the intensity of emotions. The structural model of emotions is, therefore, shaped like a cone [104]. Figure 1 demonstrates Plutchik’s wheel of emotions, biometrics and brain sensors, and trends and interdependence in this Big Picture stage. At the center of the circles is Plutchik’s wheel of emotions. Plutchik’s wheel of emotions also includes affective attitudes (interest, boredom). Plutchik [65] notes that the same instinctual source of energy is discharged as part of the emotion felt and the underlying peripheral physiological process. Emotions can be of various levels of arousal or degrees of intensity [105]. Looking at the intensity of Plutchik’s eight basic emotions, Kušen et al. [106] identified variations in emotional valence. The first circle, therefore, analyses, directly or indirectly, human arousal, valence, affective attitudes, and emotional and physiological states (AFFECT). Human AFFECT can be measured by means of neuroscience and biometric techniques. The market and global trends are a constant force affecting neuroscience and biometric technologies and their improvement. Based on the analysis of global sources [107,108,109,110] and our experience, Figure 1 presents brain and biometric sensors, as well as technique trends. Sensors will be able to integrate more and more new technologies and collect a greater variety of data, as they will become more accurate, more flexible, cheaper, smaller, greener, and more energy-efficient [108,109,110]. Network neuroscience, a new explicitly integrative approach towards brain structure and function, seeks new ways to record, map, model, and analyze what constitutes neurobiological systems and what interactions happen inside them. The computational tools and theoretical framework of modern network science, as well as the availability of new empirical tools to map extensively and record the way shifting patterns link molecules, neurons, brain areas and social systems, are two trends enabling and driving this approach [107].

**Figure 2:**
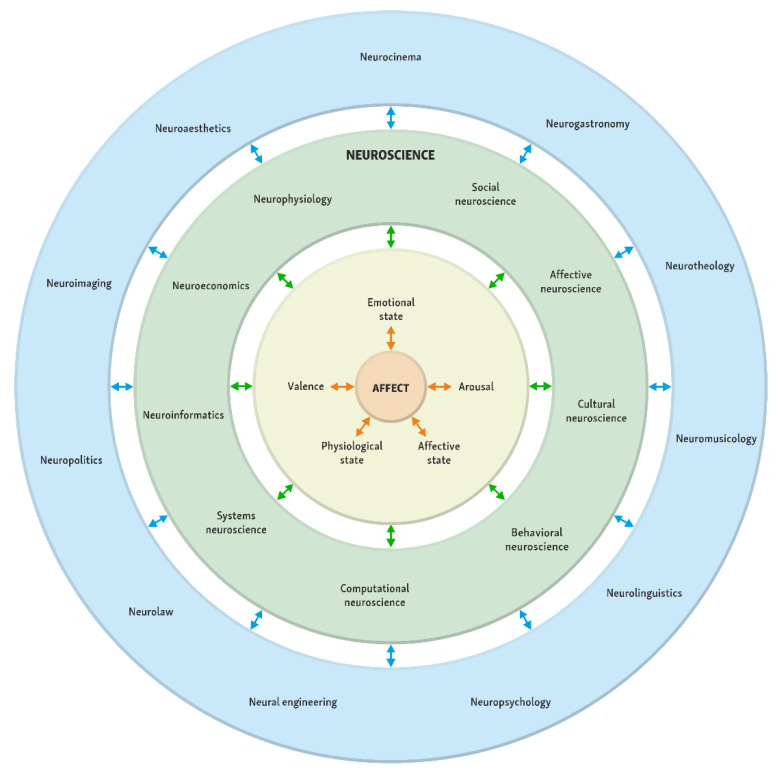
Neuroscience and biometric branches analyzing AFFECT in various sciences and fields.

Figure 2 shows numerous sciences and areas in which neuroscience and biometrics analyze the AFFECT. According to Sebastian [111], neuroeconomics is the study of the effect of anticipating money decisions on our brain. It has solidified as an entirely academic and unifying field that ventures to describe the techniques of the decision-making process; and reiterates economic behavior and decision-making process with economic disposition. The procedure of neuroeconomics involves the integration of behavioral experiments and brain imaging in order to more clearly appreciate the workings behind individual and collective decision-making [112]. Serra [113] reported that neuroeconomics researchers utilize neuroimaging devices such as functional magnetic resonance imaging (fMRI), magnetic resonance imaging (MRI), transcranial magnetic stimulation (rTMS), and transcranial direct-current stimulation (tDCS), positron emission tomography (PET) and electroencephalography (EEG). The majority of challenges probed by neuroeconomics researchers are basically similar to the problems a marketing researcher would acknowledge as aspects of their functional domain [114]. Kenning and Plassmann [115] has also defined neuroeconomics as the implementation of neuroscientific methods in the evaluation and appreciation of economically significant behavior.

According to Wirdayanti and Ghoni [116], neuromanagement entails psychology, the biological aspect of humans for decision-making in management sciences. As stated Teacu Parincu et al. [117], neuromanagement is targeted at investigating the acts of the human brain and mental performances whenever people are confronted with management challenges, using cognitive neuroscience, in addition to other scientific disciplines and technology, to evaluate economic and managerial problems. Its focal point is on neurological activities that are related to decision-making and develops personal as well as organizational intelligence (team intelligence). It also centers on the planning and management of people (for example, selection, training, group interaction and leadership) [118].

Neuro-Information Science can be defined as the science that observes neurophysiological reactions that are connected with the peripheral nervous system; that is then connected to conventional cognitive activities. Michalczyk et al. [119] stated that neuro-information-systems research has developed into a conventional approach in the information systems (IS) discipline for evaluating and appreciating user behavior. Riedl et al. [120] and Michalczyk et al. [119] concluded that Neuro-information-systems comprise studies that are centered on all types of neurophysiological techniques, such as functional magnetic resonance imaging (fMRI), electroencephalograhy (EEG), fNIRS (functional near-infrared spectroscopy), electromyography (EMG), hormone studies, or skin conductance and heart rate evaluations, as well as magnetoencephalography (MEG) and eye-tracking (ET). 

Neuro-Industrial Engineering brought about by the synergy between neuroscience and industrial engineering has afforded resolutions centered on the physiological status of people. Ma et al. [121] reported that NeuroIE secures its objective and real data by analyzing human brain and physiological indexes with advanced brain AFFECT devices and biofeedback technology, evaluating the data, adding neural activities as well as physiological status in the process of evaluation; as new constituents of operations management, and finally understanding better human–machine integration by modifying work environment and production system in line with people’s reaction to the system, preventing mishaps and enhancing efficiency and quality. According to Ma et al. [121], Neuro-Industrial Engineering is centered on humans and lays hold of human physiological status data (e.g., EEG, EMG, GSR and Temp). Zev Rymer [122] also stated that the application of Neuro-Industrial Engineering is multidisciplinary in that it cuts across the neurological sciences (particularly neurology and neurobiology) in addition to different fields of engineering disciplines such as simulation, systems modeling, robotics, signal processing, material sciences, and computer sciences. The area encompasses a range of topics and applications; for example, neurorobotics, neuroinformatics, neuroimaging, neural tissue engineering, and brain–computer interfaces.

As soon as a user contacts an insurer, a bank or any other call center, a version of Cogito’s software known as Dialog could be active in the background, assisting the client service agent to deal with the client. Should the user become upset or angry, the client service agent can ensure that necessary actions are taken to satisfy the client. According to Cogito, this service is known as “digital intuition”. Its usefulness in call centers cannot be overemphasized as it can give feedback about real-time communications. The speed at which speeches are made by the callers as well as the dynamic range of their voices can also be analyzed by the software. For example, significant variations in pitch and stresses in caller’s tones could signify excitement or anger. Less significant dynamism, a monotonous flat tone, could imply a lack of interest or unconcern. Some companies make use of the software to assist their employees engage new patients for healthcare projects that help control health challenges such as obesity or asthma. Cogito is among recent profit-based research companies whose focus are on the evaluation of signals subconsciously given off by people which exposes their mindset. The evaluation of these kinds of social-signals is beneficial beyond call centers and meeting rooms. According to Hodson [123], keeping track of conversations during surgeries or plane cockpits could assist surgeons and pilots to be aware of whether their colleagues are really attentive to their directives, possibly preserving lives.

Several areas where we can apply the technology of recognizing emotions from speech include human–computer interactions and call centers [124].

## 4. Brain and Biometric AFFECT Sensors 

### 4.1. Classifications

Globally, several classifications of biometric and neuroscience methods and technologies are used. Our research focuses on neuroscience methods that are non-invasive. The use of non-invasive brain stimulation is widespread in studies of neuroscience [125]. The non-invasive neuroscience methods are: transcranial magnetic stimulation (TMS), electroencephalography (EEG), magnetoencephalography (MEG), positron emission tomography (PET), functional magnetic resonance imaging (fMRI), near infrared spectroscopy (NIRS), diffusion tensor imaging (DTI), steady-state topography (SST), and others [126,127,128,129,130,131,132,133,134]. These non-invasive neuroscience methods are described in detail in Section 3. In the future, the authors of this article plan to analyze invasive neuroscience methods, too.

Biometrics can be physical or behavioral. In the first case, emotions can be identified by their physical features, including face, and in the second case by their behavioral characteristics, including gait, voice, signature, and typing patterns [135]. Various sensors can measure physiological signals, known as biometrics, capturing the response of bodily systems to things that are experienced through our senses, but also things imagined, by tracking sleep architecture, heart rate variability (HRV), respiratory rate (RR), and heart rate (RHR) [136].

Scientific literature classifies biometrics into certain types. Stephen and Reddy [137] and Banirostam et al. [138], for instance, classify biometrics into three categories: physiological, behavioral, and chemical/biological. Yang et al. [139] distinguish physiological and behavior traits. Kodituwakku [140] believes biometric technology can be classified into two general categories: physiological biometric techniques and behavioral biometric techniques. Jain et al. [141] and Choudhary and Naik [142] also classify biometrics into two categories: physiological and behavioral. In the literature, not only signature, voice, and gait are considered behavioral biometric features, but also ECG, EMG, and EEG [143], while other authors distinguish cognitive biometrics [144,145], including electroencephalography (EEG), electrocardiography (ECG), electrodermal response (EDR), blood pulse volume (BVP), near-infrared spectroscopy (NIR), electromyography (EMG), eye trackers (pupillometry), hemoencephalography (HEG), and related technologies [145]. Some scientific sources claim that eye tracking is a behavioral biometric [146], while others claim that it is a measurement in physiological computing [147]. Physiological biometrics measures the physiological signals to determine identity as well as authenticating and analyzing users emotions. Respiration, perspiration, heartbeat, eye-reactions to light, brain activity, emotions, and even body odor can be measured for numerous purposes, including physical and logical access control, payments, health monitoring, liveness detection, and neuromarketing among them [136].

Scientists identify the following AFFECT biometric types [139,140,141,142,148,149,150]:

- Physiological features: facial patterns, odor, pupil dilation and contraction, skin conductance, heart rate, respiratory rate, temperature, blood volume pulse, and others.
- Behavioral features: gait, keystroke, mouse tracking, signature, handwriting, speech/voice, and others.
- The authors of this article have used the classification of biometrics proposed by the abovementioned authors (physiological and behavioral features).

Biometric technologies are usually divided into those of first and second generation [151]. First-generation biometrics can confirm a person’s identity in a quick and reliable way, or authenticate them in different contexts, and law enforcement is one of the areas where such solutions are employed in practice [152]. The primary purpose of first-generation biometrics is identity verification, such as facial recognition, and the technology is built around simple sensors that capture physical features and store them for later use [153]. Second-generation biometrics can also be used to detect emotions, with electro-physiologic and behavioral biometrics (e.g., based on ECG, EEG, and EMG) as examples of such technologies [154]. Second-generation biometrics measure individual patterns of learned behavior or physiological processes, rather than physical traits, and are also known as behavioral biometrics [155]. Second-generation biometrics usage has the ability to analyze/evaluate emotions and detect intentions [156]. The use of second-generation biometrics enables wireless data collection regarding the body. The data can then be used to infer an individual’s intent and emotions, as well as emotion tracking across spaces [151,157]. We examine only physiological effects affected by emotional reactions (i.e., second-generation biometrics), and the use of biometric patterns for the identification of individuals is not discussed in this study.

A diverse range of AI algorithms have been applied for AFFECT recognition, for example machine learning, artificial neural networks, search algorithms, expert systems, evolutionary computing, natural language processing, metaheuristics, fuzzy logic, genetic algorithms, and others. Some of the most important supervised (classification, regression), unsupervised (clustering), and reinforcement learning algorithms of machine learning are common as tools in biometrics or neuroscience research to detect emotions and affective attitudes, and are listed below:

- Among classification algorithms the most common choices are: naïve Bayes [158,159,160], Decision Tree [161,162,163], Random Forest [164,165,166], Support Vector Machines [167,168,169], and K Nearest Neighbors [170,171,172].
- Among regression algorithms the usual choices are: linear regression [173,174,175], Lasso Regression [176,177], Logistic Regression [178,179,180], Multivariate Regression [181,182], and Multiple Regression Algorithm [183,184].
- Among clustering algorithms the most common choices in biometrics or neuroscience research are: K-Means Clustering [185,186,187], Fuzzy C-means Algorithm [188,189], Expectation-Maximization (EM) Algorithm [190], and Hierarchical Clustering Algorithm [188,191,192].
- Among reinforcement learning algorithms the most common choices are: deep reinforcement learning [193,194,195] and inverse reinforcement learning [196].

### 4.2. Brain AFFECT Devices and Sensors

Neuroscience is associated with multiple fields of science, for example chemistry, computation, psychology, philosophy, and linguistics. Various research areas of neuroscience include behavioral, molecular, operative, evolutionary, cellular, and therapeutic features of the neurotic system. The neuroscience market encompasses technology (electrophysiology, neuro-microscopy, whole-brain imaging, neuroproteomics analysis, animal behavior analysis, neuro-functional study, etc.), components (services, instrument, and software) and end-users (healthcare centers, research institutions and academic, diagnostic laboratories, etc.) [197]. Global Industry Analysts Inc. (San Jose, CA, USA) [197] has previously grouped the global neuroscience market into instrument, software, and services based on components.

Neuroscience provides valuable perceptions concerning the structural design of the brain and neurological, physical, and psychological activities. It helps neurologists to appreciate the various components of the brain that can assist in the development of medications and techniques to handle and avoid many neurological anomalies. The rising death rate as a result of several neurological disorders, such as Parkinson’s disease, Alzheimer’s, schizophrenia, and other brain-related health challenges, represents the basic factor controlling the neuroscience market growth [198]. According to Neuroscience Market [198], the increasing request for neuroimaging devices and the progressive brain mapping research and evaluation projects are other crucial growth-inducing factors.

Neuroscience covers a whole range of branches, such as, neuroevolution, neuroanatomy, developmental neuroscience, neuroimmunology, cellular neuroscience, neuropharmacology, clinical neuroscience, cognitive neuroscience, nanoneuroscience, molecular neuroscience, neurogenetics, neuroethology, neurochemistry, neurophysics, paleoneurobiology, neurology, and neuro-ophthalmology.

Other branches of neuroscience analyze AFFECT in various related sciences and fields, such as affective neuroscience [199,200], neuroinformatics [201,202], neuroimaging [203,204], systems neuroscience [205,206], computational neuroscience [207,208], neurophysiology [51,209], behavioral neuroscience [210,211], neural engineering [212,213], neuroeconomics [214,215], neurolinguistics [216,217], neuropsychology [218,219,220], neurophilosophy [221,222,223], neuroaesthetics [224,225,226], neurotheology [227,228,229], neuropolitics [230,231,232], neurolaw [233,234,235], social neuroscience [236,237], cultural neuroscience [238,239], neuroliterature [240,241,242], neurocinema [243,244,245], neuromusicology [246,247,248], and neurogastronomy [249,250].

For example, Lim [251] identifies the following neuroscientific techniques for neuromarketing:

- Electromagnetic methods, including magnetoencephalography (MEG), electroencephalography (EEG), and steady-state topography (SST). MEG involves the magnetic fields produced by the brain (its natural electrical currents) and is used to track the changes that occur when participants see or interact with various presentation outputs. EEG is related to the ways in which brainwaves change and is used to detect changes when participant see or interact with various promoting outputs (an electrode band or helmet is used for this purpose). SST measures a steady-state visually evoked potential, and is used to determine how brain activities change depending on the task;
- Metabolic methods, including positron emission tomography (PET) and functional magnetic resonance imaging (fMRI). PET is used to examine the metabolism of glucose within the brain with great accuracy by tracing radiation pulses, while fMRI is used to measure blood flow in the brain to determine changes in brain activity;
- Electrocardiography (ECG), which uses external skin electrodes to measure electrical changes related to cardiac cycles;
- Facial electromyography (fEMG), which amplifies tiny electrical impulses to record the physiological properties of the facial muscles;
- Transcranial Magnetic Stimulation (TMS), which is used to observe the effects of promoting output on behavior by temporarily disrupting specific brain activities. TMS is a non-invasive, safe brain stimulation method. By means of a strong electromagnet, this technique momentarily generates a short-lived virtual lesion, i.e., disrupts information processing in one of brain regions. If stimulation interferes with performing a certain task, the affected brain region is, then, necessary for normal performance of the task [252].

Table 1 demonstrates traditional non-invasive neuroscience methods.

For clarity, several descriptions of traditional neuroscience methods are presented below.

Wearable healthcare devices store a lot of sensitive personal information which makes the security of these devices very essential. Sun et al. [272] proposed an acceleration-based gait recognition method to improve gait-based elderly recognition. Gait is also a good indicator in health assessment, Majumder et al. [273] created a simple wearable gait analyzer for the elderly to support healthcare needs.

Lim [251] states that neuroscientific methods and tools include those that track, chart, and record the activity of a person’s neural system and brain in relation to a certain behavior, and neurological representations of this activity can then be generated to shed light on how an individual’s brain and nervous system respond when the person is exposed to a stimulus. In this way, neuroscientists can observe the neural processes as they happen in real time. There are three main types of neuroscientific method: those that track what is happening inside the brain (metabolic and electromagnetic activity); those that track what is happening at the neural level outside the brain; and those that can influence neural activity (Table 1, Figure 1).

Non-invasive neuroscience technical information is provided in detail in various research literature about the origin of the measured signal and the engineering/physical principle of the sensors for EEG [274,275,276], MEG [277,278,279], TMS [280,281,282], etc.

Gannouni et al. [283] have proposed a new approach with EEG signals used in emotion recognition. To achieve better emotion recognition using brain signals, Gannouni et al. [283] applied a novel adaptive channel selection method. The basis of this method is the acknowledgment that different persons have unique brain activity that also differs from one emotional state to another. Gannouni et al. [283] argue that emotion recognition using EEG signals needs a multi-disciplinary approach, encompassing areas such as psychology, engineering, neuroscience, and computer science. With the aim of improving the reproducibility of emotion measurement based on EEG, Apicella et al. [35] have proposed an emotional valence detection method for a system based on EEG, and their experiments proved an accuracy of 80.2% in cross-subject analysis and 96.1% in within-subject analysis. Dixson et al. [284] have pointed out that facial hair may interfere with detection of emotional expressions in a visual search. However, facial hair may also interfere with the detection of happy expressions within the face in the crowd paradigm, rather than facilitating an effect of anger superiority as a potential system for threat detection.

Wang et al. [285] introduced an EEG-based emotion recognition system to classify four emotion states (joy, sadness, fear, and relaxed). Their experiments used movie elicitation to acquire EEG signals from their subjects [285]. The way in which meditation influences emotional response was investigated via EEG functional connectivity of selected brain regions as the subjects experienced happiness, anger, sadness or were relaxed, before and after meditation.

Neurometrics is a quantitative EEG method. Looking at individual records, this method provides a reproducible, precise estimate of deviations from normal. Only sufficient amount of good quality raw data transformed for Gaussian distributions, correlated with age, and corrected taking into account intercorrelations among measures ensure meaningful and reliable results [286]. Businesses, government agencies, and individuals use neurometric information when they need timely and profitable decisions. Techniques based on neurometric information are applied to make profitable business decisions. These techniques are based on biometric information, eye tracking, facial action coding and implicit response testing, and are used to understand and record human sentiments and other related feedback [161].

**Figure 3:**
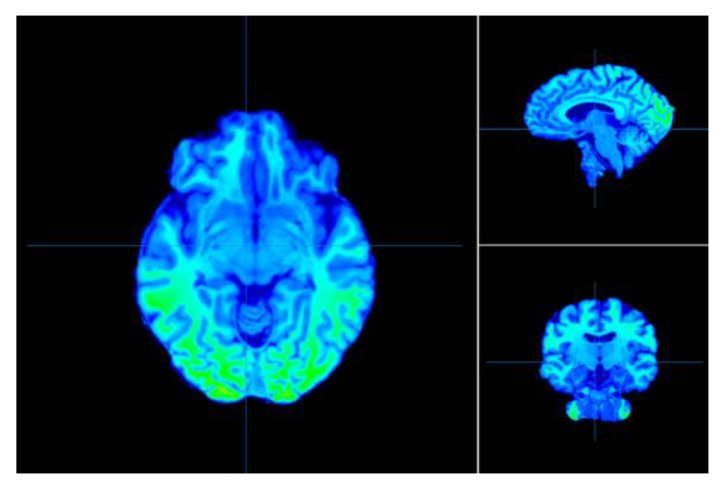
Resting state TMS brain scan image [287].

The fronto-striatal network is involved in a range of cognitive, emotional, and motor processes, such as decision-making, working memory, emotion regulation, and spatial attention. Practice shows that intermittent theta burst transcranial magnetic stimulation (iTBS) modulates the functional connectivity of brain networks. Treatments of mood disorders usually involve high stimulation intensities and long stimulation intervals in transcranial magnetic stimulation (TMS) (Figure 3) therapy [287].

**Figure 4:**
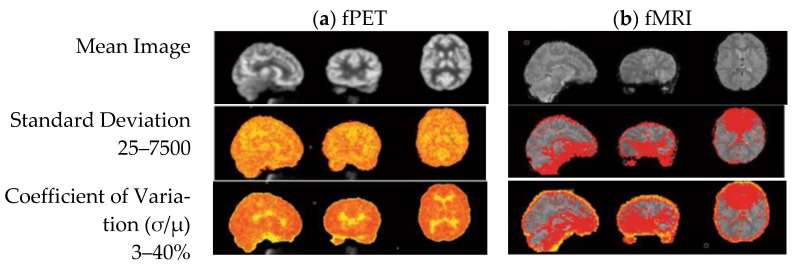
Raw images of fPET and fMRI scans [288].

One of imaging techniques is FDG-PET/fMRI (simultaneous [18F]-fluorodeoxyglucose positron emission tomography and functional magnetic resonance imaging). This technique makes it possible to image the cerebrovascular hemodynamic response and cerebral glucose uptake. These two sources of energy dynamics in the brain can provide useful information. Another greatly useful technique for characterizing interactions between distributed brain regions in humans has been resting-state fMRI connectivity, while metabolic connectivity can be a complementary measure to investigate the dynamics of the brain network. Functional PET (fPET), a new approach with high temporal resolution, can be used to measure fluoro-d-glucose (FDG) uptake and looks like a promising method to assess the dynamics of neural metabolism [288]. Figure 4 shows raw images of signal intensity variation across the brain for one individual subject.

**Figure 5:**
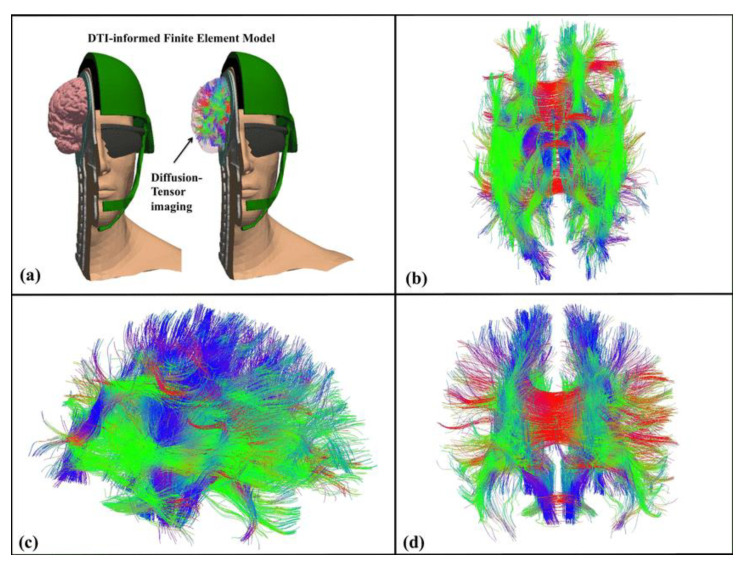
DTI can be used to construct a transversely isotropic model by overlaying axonal fiber tractography on a finite element mesh: (**a**) DTI-informed Finite Element Model; tractography shows complex fibers from (**b**) the dorsal view, (**c**) the right lateral side view, and (**d**) the posterior view. Cartography of the tracts’ position, direction by color: red for right-left, blue for foot-head, green for anterior-posterior [289].

Many biological tissues comprised of fibers, which are groups of cells aligned in a uniform direction, have anisotropic properties. In the human brain, for instance, within its white matte regions, axons usually form complex fiber tracts that enable anatomical communication and connectivity. Non-invasive tools can show the groups of axonal fibers visually. One of them is diffusion tensor magnetic resonance medical imaging (DTI), which is one particular method or application of the broader Diffusion-Weighted Imaging (DWI). The basic principle behind this technique is that water diffuses more slowly as it moves perpendicular to the preferred direction, whereas in the direction aligned with the internal structure the diffusion is more rapid. The DTI outputs can be further used to compute diffusion anisotropy measures such as the fractional anisotropy (FA). The principal direction of the diffusion tensor can also be used to obtain estimates related to the white matter connectivity in the brain. Figure 5 shows an example of DTI tractography, or visualization of the white matter connectivity [289].

### 4.3. Physiological and Behavioral Biometrics

Physiological biometrics (as opposed to behavioral biometrics) is a category of approaches that refers to physical measurements of the human body, including face, pupil constriction and dilation [290]. When a recognition system is based on physiological characteristics it can ensure a comparatively high accuracy [291]. The ubiquity of electronics such as cell phones and computers, and evolving sensor technology offer human beings new possibilities to track their behavioral and physiological features and evaluate the associated biometric results. Advances in mobile devices mean they now have many efficient and complex sensors. Biometric technology often contributes to mobile application growth, including online transaction efficiency, mobile banking, and voting. The global market for biometric systems is wide and comprises many different segments such as healthcare, transportation and logistics, security, military and defense, government, consumer electronics, and banking and finance [292].

Table 2 presents widely used physiological and behavioral biometrics.

**Figure 6:**
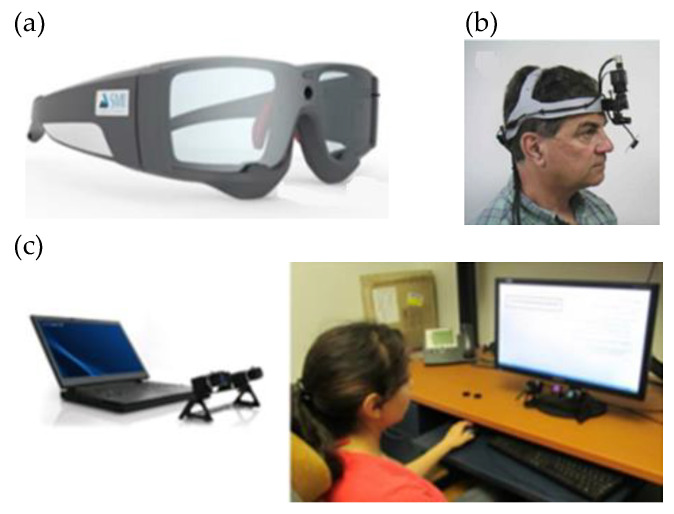
Sample of various kinds of eye-tracking tools: (**a**) eye-tracking glasses [314]; (**b**) helmet-mounted [315]; (**c**) remote or table [316].

Most of today’s eye tracking systems are video-based, with an eye video camera and infrared illumination. Eye tracking systems can be categorized as tower-mounted, mobile, or remote based on how they interface with the environment and the user (Figure 6) and different video-based eye tracking systems are required depending on the experiment, the environment, and the type of activity to be studied [313]. Researchers have used eye-tracking for behavioral research.

**Figure 7:**
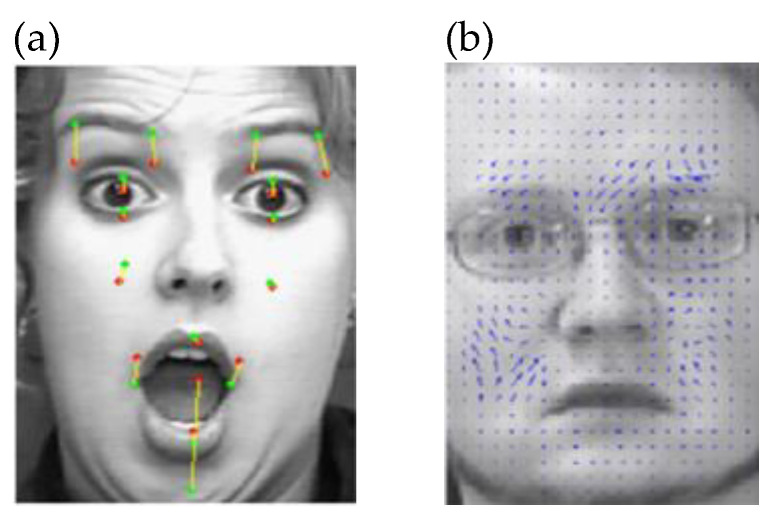
Facial expression recognition: (**a**) feature point tracking; (**b**) dense flow tracking [317].

The left image in Figure 7 shows the last frame of an expression showing surprise on a sample face from Cohn–Kanade database and highlights the trajectories (the bright lines that change color from darker to brighter from their start to end) followed by each tracked feature point. Figure 7. The application of the dense flow method (right) and the result of applying the feature optical flow on the subset of 15 points (left) [317].

**Figure 8:**
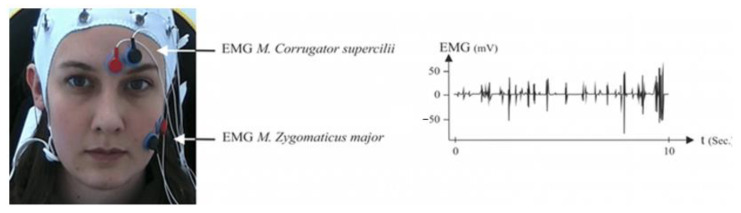
Placement of fEMG electrodes and a sample of a filtered EMG signal [319].

A group of participants were tested to record the facial EMG (fEMG) activity. Following the guidelines for fEMG placement recommended by Fridlund and Cacioppo, two 4-mm bipolar miniature silver/silver chloride (Ag/AgCl) skin electrodes were placed on their left corrugator supercilii and zygomaticus major muscle regions (Figure 7) [318]. To avoid bad signals or other unwanted influences, the BioTrace software (on NeXus-32) was used to visualize and, if necessary, correct the biosignals before each recording. Figure 8 shows the arrangement of fEMG electrodes on the M. zygomaticus major and M. corrugator supercilii. An example of a filtered electromyography (EMG) signal is shown on the right side [319].

**Figure 9:**
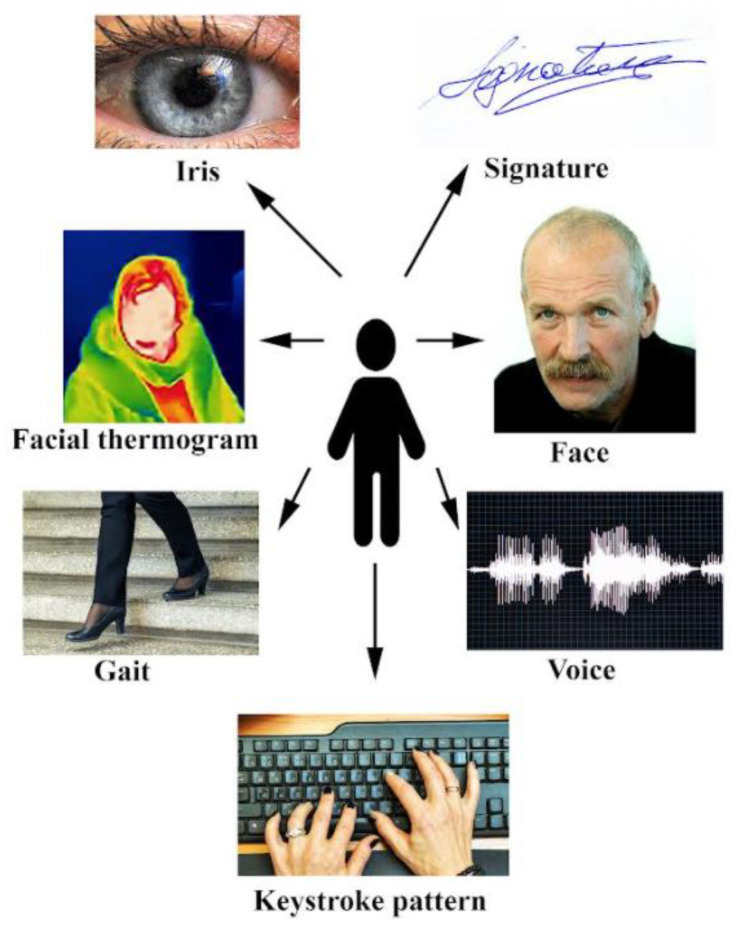
Other examples of biometric traits.

Humans have a range of biometric traits that can be a basis for various biometric recognition systems (Figure 9). The other biometrics traits are iris, face thermogram, gait, keystroke pattern, voice, face, and signature. They can have different significance. For example, iris scan has high accuracy, medium long term stability and medium security level, while voice recognition has low accuracy, low long term stability and low security level [320]. The choice of the biometric traits, however, invariably depends on the availability of the dataset’s samples, the application, the value of tolerance accepted, and the level of complexities [150].

Biometric sensors are transducers that change the biometric traits of a person, such as face, voice, and other characteristics, into an electrical signal. These sensors read or measure speed, temperature, electrical capacity, light, and other types of energy. Different technologies are available with digital cameras, sensor networks, and complex combinations. One type of sensor is required in every biometric device, and biometric sensors are a key feature of emotions recognition technology. Biometrics can be used in a microphone for voice capture or in a high-definition camera for facial recognition [321].

Jain et al. [141] state that enrolment and emotions recognition are two main phases in biometric emotions recognition systems. The enrolment phase means acquiring an individual’s biometric data to be stored in the database along with the emotions recognition details. The recognition phase uses the stored data to compare the data with the re-acquired biometric data of the same individual, to determine emotions. A biometric system is, therefore, a pattern recognition system consisting of a database, sensors, a feature extractor, and a matcher.

Loaiza [322] states that overall physiological effects related to emotional reactions depend on three types of autonomic variables: (1) the cardiac system, including blood pressure, cardiac cycles, and heart rate variability; (2) respiration, including amplitude, respiration period, and respiratory cycles; and (3) electrodermal activity, including resistance, responses, and skin conductance levels. Ekman [77] report that different emotions can have very different autonomic variables. For instance, in contrast to someone in a happy state, an angry person had a higher heart rate and temperature. Furthermore, the feeling of fear was also accompanied by higher heart rate. Pace-Schott et al. [323] argue that the ability to regulate physiological state and regulation of emotion are two inseparable features. Physiological feelings contribute to emotion regulation, reproduction, and survival.

Many works have focused on emotion detection using different techniques [35,283,284,324,325,326,327]. Specific tasks (e.g., WASSA-2017, SemEval) have also included emotion detection tasks that cover four categories of emotions (anger, fear, sadness, and joy) [320]. According to Saganowski et al. [326], the most common approach to the use of physiological signals in emotion recognition is to (1) collect and clean data; (2) to preprocess, synchronize, and integrate signal; (3) to extract and select features; and (4) to train and validate machine learning models.

Signals are a natural expression of the human body; they can be used with great success in the classification of emotional states. EEGs, temperature measurements, or electrocardiograms (ECGs) are examples of such physiological signals. They can help us to classify emotional states such as anger, sadness, or happiness, and can be captured by different sensors to identify individual differences. The goal of all of these physiological methods is to evaluate consumer attention and to obtain a particular message noticed, and their performance in this area is commendable. The advantages of these techniques include their creative and versatile placement, the stimulation of interest through novel means that capture attention, the ability to directly target and personalize messages, and lower implementation costs [328]. To study marketing trends, Singh et al. [328] recommend avoiding costly research methods such as fMRI and EEG, and instead using smaller and cheaper galvanic readings and eye tracking (ET) to investigate brain responses. These authors also propose a fuzzy rule-based algorithm to anticipate consumer behavior by detecting six facial expressions from still images. 

Various organizations are contributing to the progress of biometric standards, such as international standards organizations (International Electrotechnical Commission, ISO-JTC1/SC37, London, UK), national standards bodies (American National Standards Institute, New York, NY, USA), standards-developing organizations (International Committee for Information Technology Standards, American National Institute of Standards and Technology, Information Technology Laboratory), and other related organizations (International Biometrics and Identification Association, International Biometric Group, Biometric Consortium, Biometric Center of Excellence) [329]. De Angel et al. [330] give rise to numerous recommendations to begin improving the generalizability of the research and generating a more standardized approach to sensing in depression.

- Sample recommendations include reporting on recruitment strategies, sampling frames and participation rates; increasing the diversity of the study population by enrolling participants of different ages and ethnicities; reporting basic demographic data such as age, gender, ethnicity, and comorbidities; and measuring and reporting participant engagement and acceptability in terms of attrition rates, missing data, and/or qualitative data.
- Furthermore, in machine learning models—describing the model selection strategy, performance metrics and parameter estimates in the model with confidence intervals or nonparametric equivalents.
- Recommendations for data collection and analysis include using established and validated scales for depression assessment; presenting any available evidence on the validity and reliability of the sensor or device used; describing in sufficient detail so as to enable replication, data processing and feature construction; and providing a definition and description of how missing data is handled.
- Recommendations for data sharing include making the code used for feature extraction available within an open science framework and sharing anonymized datasets in data repositories.
- The key recommendation is recognizing the need for consistent reporting in this area. The fact that many studies—especially in the field of computer science—fail to report basic demographic information. A common framework should be developed that has standardized assessment and analysis tools and reliable feature extraction and missing data descriptions, and has been tested in more representative populations.

Neuromarketing, neuroeconomics, neuromanagement, neuro-information systems, neuro-industrial engineering, products, services, call centers studies use various instruments and techniques to measure user psychological states. Some of these tools are more complex than others, and the results that are produced can vary widely [331]. They fall into three major categories: the first two contain tools used for neuroimaging (medical devices offering in vivo information on the nervous system) and use techniques that measure brain electrical activity and neuronal metabolism, while the third contains tools used to evaluate neurophysiological indicators of the mental states of an individual. Leading neuroimaging tools such as fMRI and PET fall into the first category, while EEG, MEG, and other less invasive and cheaper neuroimaging devices that measure electrical activity in the brain [332] fall into the second category, and tools that track and record individual signals of broader physiological reaction and response measurements (e.g., electro-dermal activity, ET, etc.) fall into the third category.

Next, we overview the literature and examine the various types of arousal, valence, affective attitudes, and emotional and physiological states (AFFECT) recognition methods in more detail. A summary of the outcomes is provided in Table 3. 

The combination of several different approaches to the recognition and classification of emotional state (also known as multimodal emotion recognition) is currently a research area of great interest, especially since the use of different physiological signals can provide huge amounts of data. Since each physiological can make a significant impact on the ability to classify emotions [333]. Table 3 presents an overview of studies related to the recognition of valence, arousal, emotional states, physiological states, and affective attitudes (affect). A brief overview of some of these studies follows.

Many scientists and practitioners have earned acclaim and honor for their research in areas such as diagnostics, large-scale screening, analysis, monitoring, and categorizations of people by COVID-19 symptoms. Their work relied on early warning systems, wearable technologies, the Internet of Medical Things, IoT based systems, biometric monitoring technologies, and other tools that can assist in the COVID-19 pandemic. Javaid et al. [438] review how different industry 4.0 technologies (e.g., AI, IoT, Big data, Virtual Reality, etc.) can help reduce the spread of disease. Kalhori et al. [439] and Rahman et al. [440] discuss the digital health tools to fight COVID-19. Various sensors and mobile devices to detect the disease, reduce its spread, and measure different symptoms are also widely discussed. Rajeesh Kumar et al. [441] propose a system to identify asymptotic patients using IoT-based sensors, measuring blood oxygen level, body temperature, blood pressure, and heartbeat. Stojanović et al. [442] propose a phone headset to collect information about respiratory rate and cough, Xian et al. [443] present a portable biosensor to test saliva. Chamberlain et al. [444] presented distributed networks of Smart thermometers track COVID-19 transmission epicenters in real-time.

Neurotransmitters (NT) are billions of molecules constantly needed to keep human brains functioning. They are chemical messengers that carry, balance, and boost signals travelling between nerve cells (neurons) and other cells in the body. Many different psychological and physical functions can be affected by these chemical messengers, including fear, appetite, mood, sleep, heart rate, breathing rate, concentration and learning [445]. Lim [251] has also outlined new ways of exploiting neuromarketing research to achieve a better understanding of the brain and neural activity and hence advance marketing science. Lim [251] highlighted three main aspects: (i) antecedents (such as the product, physical evidence, the price of the product, the place where everything is happening, promotion, the process involved, people); (ii) the process; and (iii) the consequences for the target market (behavioral outcomes before, during and after the act of buying) and the marketing organization (visits, sales, awareness, equity). Agarwal and Xavier [253] described the most popular neuromarketing tools, including event-related potential (ERP) (P300), EEG, and fMRI, and explained how these tools could be applied in marketing. A business and marketing article [256] lists the three categories of neuroscientific techniques that are applied in business and advertising research (Table 1 and Table 2, Figure 1) as follows:

1. Methods that monitor what is happening in the brain (i.e., the physiological activity of the CNS);
2. Methods that record what is happening elsewhere in the body (i.e., the physiological activity of the PNS);
3. Other techniques for tracking behavior and conduct.

Ganapathy [260] groups neuromarketing tools into three categories (Table 1 and Table 2). Farnsworth [258] gives information that can be essential when deciding on the best neuromarketing method or technique to help stakeholders understand research methods relating to human behavior at a glance, while Saltini [264] gives a short list of neuromarketing tools (Table 1 and Table 2). A system developed by CoolTool [257] allows several neuromarketing tools to be used separately or combined. 

Although individual neuroscientific tools for neuromarketing, neuroeconomics, neuromanagement, neuro-information systems, neuro-industrial engineering, products, services, call centers have been developed by many researchers (for example [111,251,253,254,255,256,257,258,259,260,261,262,263,264,265,266,267,268,269,270,293,298,299,300,303,309,311,312,328,446,447,448], a review and analysis of the complete range of tools used in neuromarketing, neuroeconomics, neuromanagement, neuro-information systems, neuro-industrial engineering, products, services, call centers research has not yet been carried out. Thorough examinations of the range of research tool alternatives that are available for neuroscience are also often missing from research in this area. We have therefore compiled a complete list of neuroscience techniques for neuromarketing, neuroeconomics, neuromanagement, neuro-information systems, neuro-industrial engineering, products, services, call centers. Humans experience emotions and their associated feelings (e.g., gratitude, curiosity, fear, sadness, disgust, happiness, and pride) on a daily basis. Yet, in case of affective disorders such as depression and anxiety, emotions can become destructive. Thus the focus on understanding emotional responsiveness is not surprising in neuroscience and psychological science [449]. So neuroscience techniques analyze emotional, affective and physiological states tracking neural/electrical activity [335,336,337,338,339,340,450,451] or neural/metabolic activity [341,342,343,344,349,447,452,453] within the brain. This is also presented in Table 3.

For example, neuromarketing techniques can complement business decisions and make them more profitable, using the automated mining of opinions, attitudes, emotions and expressions from speech, text, emotions, neuron activity and other database-fed sources. Advertisements that are adjusted based on such information can engage the target audience more effectively and make a better impact on the audience, and this may translate into better sales and higher margins. In an attempt to enhance corporate branding and advertising routines, various factors have been studied, such as emotional appeal and sensory branding, to ensure that companies deliver the right message and that customers perceive the right message [171].

Affect recognition is widely used in gaming to create affect-aware video games and other software. Alhargan et al. [454] present affect recognition in an interactive gaming environment using eye-tracking. Szwoch and Szwoch [455] give a review of automatic multimodal affect recognition of facial expressions and emotions. Krol et al. [456] combined eye-tracking and brain–computer interface (BCI) and created a completely hands-free game Tetris clone where traditional actions (i.e., block manipulation) are performed using gaze control. Elor et al. [457] measure heart rate and galvanic skin response (GSR) with Immersive Virtual Reality (iVR) Head-Mounted Display (HMD) systems paired with exercise games to show how exercise games can positively affect physical rehabilitation.

Stress is a relevant health problem among students, so Tiwari, Agarwal [458] present a stress analysis system to detect stressful conditions of the student, including measurement of GSR and electrocardiogram (ECG) data. Nakayama et al. [459] suggest measuring heart rate variability as a method to evaluate nursing students stress during simulation to provide a better way to learn.

A literature review can reveal the most popular types of traditional and non-traditional neuromarketing methods. According to Sebastian [111], focus groups are one of the more traditional marketing methods, while various neuroscience techniques have also been applied to record the metabolic activity of the body and the electrical activity of the brain (transcranial magnetic stimulation (TMS), electroencephalography (EEG), functional magnetic resonance imaging, magnetoencephalography (MEG), and positron-emission tomography (PET)).

Electronic platforms are not the only possibility for non-traditional marketing, and Tautchin and Dussome [460] believe that traditional media can also be reimagined in new forms, such as guerrilla marketing, local displays, vehicle wraps, scaffolding, and even bubble cloud ads or aerial banners. In addition to giving high-quality feedback data, non-traditional techniques can also help in the evaluation of business decisions and conclusions [328]. 

Based on factors such as skin texture, gender, and SC, wearable biometric GSR sensors could be used to identify whether a person is in a sad, neutral, or happy emotional state. To understand marketing strategies better and to improve ads, other biometric sensors such as pulse oximeters and health bands could be used in the future to make automated predictions of emotions [461]. The galvanic skin response (GSR) method has an important limitation—it does not provide information on valence. The usual way to address this issue is to use other emotion recognition methods. They provide additional details and thus enable detailed analysis. Table 3 lists studies where GSR is used to measure emotions. 

Eye tracking (ET) is used to record the frequencies of choices; sensor features are extracted and matched with certain preference labels to determine mutual dependences and to discover which brain regions are active when a certain choice task is performed. High values for alpha, beta and theta waves have been reported in the occipital and frontal brain regions, with a high degree of synchronization. A hidden Markov model is a popular tool for time-series data modeling, and researchers have successfully used this approach to build brain–computer-interface tools with EEG signals, counting mental task classification, medical applications and eye movement tracking [462].

A classification model based on SVM architecture, developed by Lakhan et al. [463], can predict the level of arousal and valence in recorded EEG data. Its core is a feature extraction algorithm based on power spectral density (PSD).

Multimodal frameworks that combine several modalities to improve results have recently become popular in the domain of human–computer interaction. A combination of modalities can give a more efficient user experience since the strengths of one modality can offset the weaknesses of another and the usability can be increased. These systems recognize and combine different inputs, taking into account certain contextual and temporal constraints and thus facilitating interpretation. Kong et al. [464] created a way of using two different sensors and calibrating them to achieve simultaneous gesture recording. Hidden Markov Model (HMM) was used for all single- and double-handed gesture recognition. Multimodality means that several unimodal solutions are combined into a system, meaning that multiple solutions can be combined into a single best solution using optimization algorithms [464].

The automatic emotion recognition system proposed by El-Amir et al. [465] uses a combination of four fractal dimensions and detrended fluctuation analysis, and is based on three bio-signals, GSR, EMG, and EEG. Using two emotional dimensions, the signals were passed to three supervised classifiers and assigned to three different emotional groups, with a maximum accuracy for the valence dimension of 94.3% and a maximum accuracy for the arousal dimension of 94%. This approach is based on external signals such as facial expressions and speech recognition, which means that it is simple and that no special equipment is required. The limitations of this approach are that emotions can be faked, and that these types of recognition methods fail with disabled people and people with certain diseases. Other approaches are based on electromyography, ECGs, SC, EEGs, and other physiological signals that are spontaneous and cannot be consciously controlled [465].

Plassmann et al. [466] as well as Perrachione and Perrachhione [467] carried out exciting studies in an attempt to determine how marketing stimuli lead to buying decisions. They applied neurosciences to marketing in order to create better models and to understand of how a buyer’s brain and emotions operate. Gruter [468] states that a wide range of techniques and tools are used to measure consumer responses and behavior. Three approaches that are used in neuromarketing can give access to the brain: input and output models, internal reflexes, and external reflexes. 

Leon et al. [469] present a real-time recognition and classification method based on physiological signals to track and detect changes in emotions from a neutral state to either a positive or negative (i.e., non-neutral) state. They used the residual values of auto-associative neural networks and the statistical probability ratio test in their approach. When the proposed methodology was implemented to process a recognition level of 71.4% was achieved [469]. Monajati et al. [470] also investigated the recognition of negative emotional states, using the three physiological signals of galvanic skin response, respiratory rate and heart rate. Fuzzy-ART was applied to analyze the physiological responses and to recognize negative emotions. An overall accuracy of 94% was achieved in determining which emotions were negative as opposed to neutral [470].

Andrew et al. [471] described investigations of brain responses to modern outdoor advertising, focusing on memorability, visual attention, desirability, and emotional intensity. They also described ways in which the latest imaging tools and methods could be applied to monitor subconscious emotional responses to outdoor media in many forms, from multisensory advertising screens to simple paper posters. Andrew et al. [471] explained the cognitive processes behind their success, not solely in the context of the advertising to which people are typically exposed outside their homes, but also in the broader digital world. Andrew et al. findings have fundamental implications for media campaign planning, design, and development, identifying the possible role of outdoor advertising compared to other media, and possible ways of combining different media platforms and making them work for the benefit of advertisers.

Kaklauskas et al. [472] integrated Damasio’s somatic marker hypothesis with biometric systems, multi-criteria analysis techniques, statistical investigation, a neuro-questionnaire, and intelligent systems to produce the INVAR neuromarketing system and method. INVAR can measure the efficiency of both a complete video advertisement and its separate frames. This system can also determine which frames make viewers interested, confused, disgusted, happy, scared, surprised, angry, sad, bored, or confused; can identify the utmost positive or negative video advertisement; measure the consequence of a video advertisement on long-term and short-term memory; and perform other functions. 

Lajante and Ladhari [473] applied peripheral psychophysiology measures in their research, based on the assumption that measures of emotion and cognition such as SC responses and facial EMGs could make a significant contribution to new ideas about consumer decision making, judgments and behaviors. These authors believe that their approach can help in applying affective neuroscience to the field of consumer services and retailing.

Michael et al. [474] aimed to understand the ways in which unconscious and direct cognitive and emotional responses underlie preferences for particular travel destinations. A 3×5 factorial design was run in order to better understand the unconscious responses of consumers to possible travel destinations. The factors considered in this study were the type of stimulus (videos, printed names, and images) and the travel destination (New York, London, Hong Kong, Abu Dhabi, and Dubai). ET can provide reliable tracking of cognitive and emotional responses over time. The authors suggested that decisions on travel destinations have both a direct and an unconscious component, which may affect or drive overt preferences and actual choices. 

Harris et al. [448] investigated ways of measuring the effectiveness of social ads of the emotion/action type, and then of making these ads more effective using consumer neuroscience. Their research offers insights into changes in behavioral intent brought about by effective ads and gives an improved understanding of ways of making good use of social messages regarding a certain action, challenge or emotion that may be needed to help save lives. It can also reduce spending on social marketing campaigns that end up being ineffectual.

Libert and Van Hulle [475] argue that the development of economically practicable solutions involving human–machine interactions (HMI) and mental state monitoring, and neuromarketing that can benefit severely disabled patients has put brain–computer interfacing (BCI) in the spotlight. The monitoring of a customer’s mental state in response to watching an ad is interesting, at least from the perspective of neuromarketing managers. The authors propose a method of monitoring EEGs and predicting whether a viewer will show interest in watching a video trailer or will show no interest, skipping it prematurely. They also trained a k-nearest neighbor (kNN), a support vector machine (SVM), and a random forest (RF) classifier to carry out the prediction task. The average single-subject classification accuracy of the model was as follows: 73.3% for viewer interest and 75.803% for skipping using SVM; 78.333% for viewer interest and 82.223% for skipping using kNN; and 75.555% for interest and 80.003% for skipping using RF.

Jiménez-Marín et al. [476] showed that sensory marketing tends to accumulate user experiences and then exploit them to bring the users closer to the product they are evaluating, thus motivating the final purchase. However, several issues need to be considered when these techniques are applied to reach the desired outcomes, and it is important to be aware of recent advances in neuroscience. The authors explore the concept of sensory marketing, pointing out its possibilities for application and its various typologies. 

Cherubino et al. [477] highlighted the new technological advances that have been achieved over the last decade, which mean that research settings are now not the only scenarios in which neurophysiological measures can be employed and that it is possible to study human behavior in everyday situations. Their review aimed to discover effective ways to employ neuroscience technologies to gain better insights into human behavior related to decision making in real-life situations, and to determine whether such applications are possible. 

Monica et al. [478] explored the cognitive understanding and usability of banking web pages. They reviewed the theoretical literature on user experience in online banking services research, with a focus on ET as a research tool, and then selected two Romanian banking websites to study consumer attention, while consumers were navigating the sites, and memory, after their visits. The research findings showed that the layout and information display can make web pages more or less usable and can have an effect on cognitive understanding.

Singh et al. [328] discussed various methods of feature extraction for facial emotion detection. The algorithm they proposed could detect a total of six facial emotions, using a fuzzy rule-based system. During their experiment, neurometrics were recorded using a system comprising MegaMatcher software, Grove-GSR Sensor V1.2, and a 12-megapixel Hikvision IP camera. The participants were asked to watch a set of video ads for a range of well-known cosmetic products and wore SC sensors and sat in front of a camera that monitored their responses. Singh et al. [328] analyzed the cognitive processes of university students in relation to advertising and compliance with the code of self-regulation. A quantitative and qualitative methodology based on facial expressions, ET techniques and focus groups was used for this purpose. The results suggested that online game operators could be clearly identified. A high interaction of the public within the exhibition of supposed skills of the successful player and welcome bonuses also exists, and there was shown to be a lack of knowledge of the visual elements of awareness, a trivialization of compulsive gambling, and sexist attitudes towards women attracting public attention. A positive public attitude towards gaming was also observed by Singh et al. [328]; it was seen as a healthy form of leisure that was compatible with family and social relationships.

Goyal and Singh [461] proposed the use of research-based approaches for the automatic recognition of human affective facial expressions. These authors created an intelligent neural network-based system for the classification of expressions from extracted facial images. Several basic and specialized neural networks for the detection of facial expressions were used for image extraction. 

Electromyography measures and assesses electric potentials in muscle cells. In medical settings, this method is used to identify nerve and muscle lesions, while in emotion recognition this method is used to look for correlations between emotions and physiological responses. Most EMG-based studies examine facial expressions drawing on the hypothesis that facial expressions take part in emotional responses to various stimuli. The hypothesis was first proposed by Ekman and Friesen in 1978; they described the relationships between basic emotions, facial muscles, and the actions they trigger. Morillo et al. [479] used low-cost EEG headsets and applied discrete classification techniques to analyze scores given by subjects to individual TV ads, using artificial neural networks, the C4.5 algorithm and the Ameva discretization algorithm. A sample of 1400 effective advertising campaigns was studied by Pringle et al. [480], who determined that promotions with exclusively emotional content achieved around double (31% vs. 16%) success as those with only rational content, while compared to campaigns with mixed emotional and rational content, the exclusively emotional campaigns performed only slightly better (31% vs. 26%). 

According to Takahashi [481] some of the available emotion recognition systems in facial expressions or speech look at several emotional states such as fear, teasing, sadness, joy, surprise, anger, disgust, and neutral. Takahashi [481] investigated emotion recognition based on five emotional states (fear, anger, sadness, joy, and relaxed).

The authors [353,355,356,357,359,360,371,372,373,374] carried out an in-depth analysis of how blood pressure, SC, heart rate and body temperature depend on stress and emotions. Figures suggest that work-related stress costs the EU countries at least EUR 20 billion annually. Stress experienced at work can cause anxiety, depression, heart disease and increased chronic fatigue which can have a considerable negative impact on creativity, competitiveness and work productivity.

Research worldwide shows that people exposed to stress can experience higher blood pressure and heart rate. Light et al. [482] analyzed cases of daily elevated stress levels and looked at the effects on fluctuations in systolic and diastolic blood pressure. Gray et al. [483] investigated how systolic and diastolic blood pressure can be affected by psychological stress, while Adrogué and Madias [484] described the effects of chronic, emotional and psychological stress on blood pressure. The unanimous conclusion of research in this area is that diastolic and systolic blood pressure and heart rate depend on stress and can increase depending on the level of stress.

Blair et al. [485] analyzed the effect of stress on heart rate and concluded that heart rate rises sharply within three minutes of the onset of stress and starts to fall only after another five to six minutes. Gasperin et al. [486] concluded that high blood pressure was affected by chronic stress. A number of studies have shown that patients with heart rates higher than 70 beats per minute are more likely to develop cardiovascular diseases and to die from them; tests show that a rapid heartbeat increases the risk of heart attack by 46%, heart insufficiency by 56% and death by 34%.

Sun et al. [487] proposed an activity-aware detection scheme for mental stress. Twenty participants took part in their experiment, and galvanic skin response, ECG, and accelerometer data were recorded while they were sitting, standing, and walking. Baseline physiological measurements were first taken for each activity, and then for participants exposed to mental stressors. The accelerometer was used to track activity, and the data gave a classification accuracy between subjects of 80.9%, while the 10-fold cross-validation accuracy for the classification of mental stress reached 92.4%. This study focused on physiological signals for example photoplethysmography and galvanic skin response. The neural network configurations (both recurrent and feed forward) were examined and a comprehensive performance analysis showed that the best option for stress level detection was layer recurrent neural networks. For a sample of 19 automotive drivers, this evaluation achieved an average sensitivity of 88.83%, a precision of 89.23% and a specificity of 94.92% [488].

Palacios et al. [489] applied a new process involving two databases containing utterances under stress by men and women. Four classification methods were used to identify these utterances and to organize them into groups. The methods were then compared in terms of their final scores and quality performance.

Fever occurs when the body’s thermoregulatory set point increases, and many findings suggest that the rise in core temperature induced by psychological stress can be seen as fever. A fever of psychological origin in humans might then be a result of this mechanism [490].

Wu and Liang [491] presented a training and testing procedure for emotion recognition based on semantic labels, acoustic prosodic information and personality traits. A recognition process based on semantic labels was applied, using a speech recognizer to identify word sequences, and HowNet, a Chinese knowledge base, was used as the source for deriving the semantic word sequence labels. The emotion association rules (EARs) of the word sequences were then mined by applying a text-based mining method, and the relationships between the EARs and emotional states were characterized using the MaxEnt model. In a second approach based on acoustic prosodic information, emotional salient segments (ESSs) were detected in utterances and their prosodic and acoustic features were extracted, including pitch-related, formant, and spectrum attributes. The next step was the construction of base-level classifiers using SVM, gaussian mixture models (GMM) and MLP, which were then combined (using MDT) by selecting the most promising option for emotion recognition based on acoustic prosodic information. The process ended when the final emotional state was determined. A weighted product fusion method was applied to combine the outputs produced by the two types of recognizers. The personality traits of the specific speaker, as determined from the Eysenck personality questionnaire, were then taken into consideration to examine their impact and personalize the emotion recognition scheme [491].

A hybrid analysis method for online reviews proposed by Nilashi et al. [492] allows for the ranking of factors affecting the decisions of travelers in their choice of green hotels with spa services. This method combined text mining, predictive learning techniques and multiple criteria decision-making methods, and was proposed for the first time in the context of hospitality and tourism, with an emphasis on green hotel customer grouping based on online customer feedback. Nilashi et al. [492] used the latent Dirichlet analysis method to analyze textual reviews, a self-organizing map for cluster analysis, the neuro-fuzzy method to measure customer satisfaction, and the TOPSIS method to rank the features of hotels. The proposed method was tested by analyzing travelers’ reviews of 152 Malaysian hotels. The findings of this research offer an important method of hotel selection by travelers, by means of user-generated content (UGC), while hotel managers can use this approach to improve their marketing strategies and service quality.

A neuromarketing method for green, energy-efficient and multisensory homes, proposed by Kaklauskas et al. [493], can be used to determine the conditions that are required. The multisensory dataset (physiological and emotional states) collected as part of this research contained about 200 million data points, and the analysis also included noise pollution and outdoor air pollution (volatile organic compounds, CO, NO_2_, and PM10). This article discussed specific case studies of energy-efficient and green buildings as a demonstration of the proposed method. The results matched findings from both current and previous studies, showing that the correlation between age and environmental responsiveness has an inverse U shape and that age is an important factor affecting interest in eco-friendly, energy-efficient homes.

The VINERS method and biometric techniques developed by Kaklauskas et al. [494] for the analysis of emotional states, physiological reactions and affective attitudes were used to determine which locations are the best choice and then to show neuro ads of available homes offered for sale. Homebuyers were grouped into rational segments, taking into account consumer psychographics and behavior (happy, angry or sad, and valence and heart rate) and their demographic profiles (age, gender, marital status, children or no children, education, main source of income). A rational video ad for the respective rational segment was then selected. This study aimed to combine the somatic marker hypothesis, neuromarketing, biometrics and the COPRAS method, and to develop the VINERS method for use with multi-criteria analysis and the neuromarketing of the best places to live. The case study presented in the article demonstrated the VINERS method in practice. 

Etzold et al. [495] examined the case of users booking appointments online, and the ways in which they interacted with the webpage interface and visualizations. The main point was to determine whether a new interface for online booking was easy to navigate and successful in attracting user attention. In this study, the authors particularly wanted to determine whether a new, more expensive customer website was seen as more user-friendly and supportive than the older, cheaper alternative. An empirical study was carried out by tracking users eye movements as they were navigating the existing website of Mercedes-Benz, a car manufacturer, and then a new, updated version of the same company’s website. A total of 20 people were observed, and evaluations of their ET data suggested that the new service appointment booking interface could be further improved. Scan-paths and heatmaps demonstrated that the old website was superior [495]. 

In recent years, many different emotional values, such as the net emotional value (NEV), the service encounter emotional value (SEEVal), and others, have been analyzed. Attempts have been also made to put them into practice [496,497,498,499,500,501,502,503]. These studies are overviewed below. To calculate NEV, the average score for negative emotions (stressed, dissatisfied, frustrated, unhappy, irritated, hurried, disappointed, neglected) is subtracted from the average score for positive emotions (cared for, stimulated, happy, pleased, trusting, valued, focused, safe, interested, indulgent, energetic, exploratory). The average score obtained this way can be used to characterize a client’s feelings about a service or a product [499]. A higher value of NEV indicates that the relationships forged by a business are more reliable. One advantage of the NEV is that it characterizes the total balance of a consumer’s feelings related to products or services, and thus reveals the value drivers. The relationship between NEV and client satisfaction is linear [500].

The NEV can be used to highlight both aspects that need to be improved, and those that are positive. Since the NEV is calculated based on a subtraction, the result may be either a negative or a positive number. The overall score can indicate what is happening with the client at an emotional level, and suggest ways to use this to gain competitive advantage [501]. 

The SEEVal is another measure proposed by Bailey et al. [504], and is the sum of the NEV experienced by the client and the NEV experienced by the product or service provider’s employee. The client’s end results linked to SEEVal are typically loyalty, satisfaction, pleasure, and voluntary benevolence [504]. The IGI Global Dictionary defines an emotional value as a set of positive moods (feeling good or being happy) resulting from products or services and contained in the value gain from the customers’ emotional states or feelings when using the products or services (IGI Global Dictionary). Emotional value acts as a moderator, and has significant effects on the roles of social, functional, epistemic, conditional and environmental values [497]. 

Zavadskas et al. [505] examined data on potential buyers to analyze the hedonic value in one-to-one marketing situations. They used the neutrosophic PROMETHEE technique to examine arousal, valence, affective attitudes, emotional and physiological states (AFFECT), and argued that hedonic value is tied to several factors including customers’ social and psychological data, client satisfaction, criteria of attractiveness, aesthetics, and economy, the sales site rental price, emotional factors, and indicators of the purchasing process. Their research showed that an analysis of the aforementioned data on potential buyers can make an important contribution to more effective one-to-one marketing. The case study cited in this work concerned two sites in Vilnius and intended to calculate the hedonic value of these sites during the Kaziukas Fair.

**Figure 10:**
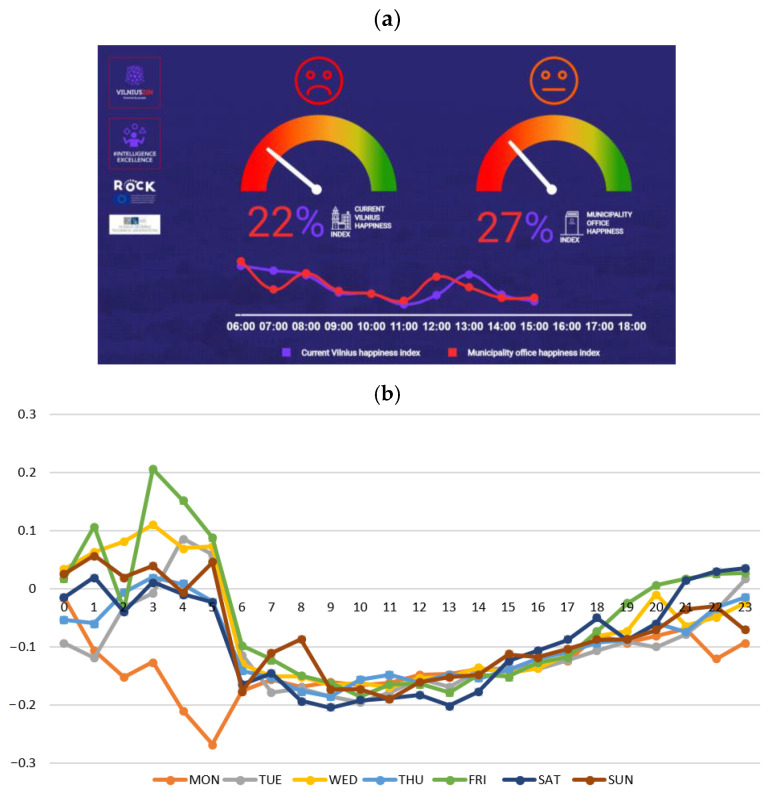
Real-time Vilnius Happiness Index (**a**) and the mean magnitudes of valence, by the hour, on weekdays (**b**).

The ROCK Video Neuroanalytics and associated e-infrastructure were established as part of the H2020 ROCK project. This project tracked passers-by at ten locations across Vilnius. One of our outputs is the real-time Vilnius Happiness Index (Figure 10 and https://api.vilnius.lt/happiness-index, accessed on 5 September 2022). The project also involved a number of additional actions (https://Vilnius.lt/en/category/rock-project/, accessed on 5 September 2022).

The intensity of the most intense negative emotion (scared, disgusted, sad, angry) subtracted from the intensity of “happiness” equals valence [430]. This way the single score of valence combines both positive and negative emotions. Our pool of data comprised 208 million data points analyzed using SPSS Statistics, a statistical software suite. Figure 10b presents the average values of valence per hour on weekdays. Every hour, the changes of average valence among Vilnius passers-by were recorded. Valence was measured every second and these values were accumulated by weekdays (marked in the chart with specific colors) at 95% confidence intervals. The y-axis shows the average values of valence (which fluctuates between −1 to 1) for each full day, for seven days, and the x-axis shows the hour starting at midnight [348].

## 5. Users’ Demographic and Cultural Background, Socioeconomic Status, Diversity Attitudes, and Context

Emotions are a means to engage in a relationship with others: Anger means that the person refuses to accept a specific treatment from others and expresses that they feel entitled to something more. Anger is expressed with the aim of influencing, controlling, and fixing the behavior of others [506].

Through emotions, people can adaptively respond to opportunities and demands they face around them [507,508,509]. When people face everyday stressors, stressful transitions, ongoing challenges, and acute crises, the adaptive function of emotions is evident in all of these situations. Emotions also depend on context [510]. This means that emotions are most effective when people express them in the situational contexts for which the emotions most likely evolved. In addition, they are specifically most likely to promote adaptation in such scenarios. The experience of anger, for instance, is adaptive because it motivates the focus of energies and the mobilization of resources toward an effective response. When a person expresses anger, adaptive mechanisms are also at work because it shows the person’s willingness, and perhaps even ability, to defend themselves. Emotional responses are sensitive to contexts, and are therefore, an integral part of our ways to adapt to daily life and the environment [511].

The ability to modify emotion responses according to changing context may be an important element of psychological adjustment [510]. An individual’s capacity to modify emotion responses taking into account the demands of changing contexts (i.e., environmental or interpersonal) is particularly relevant. This mechanism is known as emotion context sensitivity [511].

Cultural and gender differences in emotional experiences have been identified in previous research [512]. For instance, these authors used the Granger causality test to establish how a person’s cultural background and situation affect emotion. The conclusions drawn by [513] propose a top-down mechanism where gender and age can impact the brain mechanisms behind emotive imagery, either directly or by interacting with bottom-up stimuli.

Cultural neuroscientists are studying how cultural traits such as values, beliefs, and practices shape human affective, emotional, and physiological states (AFFECT) and behavior. Hampton and Varnum [514] have reviewed theoretical accounts on how culture impacts internal experiences and outward expressions of emotion, as well as how people opt to regulate them. They also analyze cultural neuroscience research that investigates how emotion regulation varies in different cultural groups.

Thus far, differences between nations have largely been the focus in studies of culture in social neuroscience. Culture impacts more than just our behavior—it also plays a role in how we see and interpret the world [515]. For instance, socioeconomic factors such as education, occupation, and income have a significant impact on how a person thinks. In one study, working-class Americans were shown to exhibit a more context-dependent thought process, similar to the collectivist patterns seen in other countries. Individuals of a lower social class in terms of their socio-economic status agreed with contextual explanations of economic trends, broad social outcomes, and emotions [516].

Gallo and Matthews [517] looked at the indirect evidence that socioeconomic status is associated with negative emotions and cognition, and that negative emotions and cognition are associated with target health status. They also proposed a general framework for understanding the roles of cognitive–emotional factors, arguing that low socioeconomic status causes stress, and impairs a person’s reserve capacity for managing it, thus heightening emotional and cognitive vulnerability. 

Choudhury et al. [518] explore critical neuroscience, a field of inquiry that probes the social, cultural, political, and economic contexts and assumptions that form the basis for behavioral and brain science research. 

Numerous studies have illustrated that depending on the specific demographic background, there are major differences between users’ emotions, behavior, and perceived usability. According to Goldfarb and Brown [519], scientific research is characterized by racial, cultural, and socioeconomic prejudices, which lead to demographic homogeneity in participation. This in turn spurs inaccurate representations of neurological normalcy and leads to poor replication and generalization. 

According to Freud, the unconscious is a depository for socially unacceptable ideas, wishes or desires, traumatic memories, and painful emotions that psychological repression had pushed out of consciousness [520]. HireVue, which is a global front-runner in AI technologies, is one of the top emotional AI companies that is now turning to biosensors that read non-conscious data in lieu of facial coding methods to measure emotions [521].

The ideas of what it means to have good relationships and to be a good person differ in different cultural contexts [522]. People’s emotional lives are closely related to these different ideas of how people see themselves and their relationships: Emotions usually match the cultural model [523,524]. Therefore, rather than being random, cultural variation in emotions matches the cultural ideals of ways to be a good person and to maintain good relationships with other people [506].

Aside from being biologically driven, emotion is also influenced by environment, as well as cultural or social situations. Culture can constrain or enhance the way emotions are felt and are expressed in different cultural contexts, and it can influence emotions in other ways. Studies have consistently shown cross-cultural differences in the levels of emotional arousal. Eastern culture, for instance, is related to low arousal emotions, whereas Western culture is related to high arousal emotions [525]. Many findings in cross-cultural research suggest that decoding rules and cultural norms influence the perception of anger [526]. Scollon et al. [527] look at five cultures (Asian American, European American, Hispanic, Indian, and Japanese) to assesses the way emotions are experienced in these cultures. Pride shows the greatest cultural differences [527]. As emotions are fundamentally genetically determined, different ones are perceived in similar ways throughout most nations or cultures [528].

## 6. Results

The present article aims to bridge the affective biometrics and neuroscience gap in existing knowledge, in order to contribute to the overall knowledge in this area. We also aim to provide information on the knowledge gaps in this area and to chart directions for future research. 

We conclude this review by discussing unanswered questions related to the next generation of AFFECT detection techniques that use brain and biometric sensors.

By performing text analytics of 21,397 articles that were indexed by Web of Science from 1990 to 2022, we examined the key changes in this area within the last 32 years. Scientific output relating to AFFECT detection techniques using brain and biometric sensors is steadily increasing. As this trend suggests, there has been continuous growth in the number of papers published in the field, with the total number of articles appearing between 2015 and 2021 nearing the total number of articles published over the previous 25 years (1990 to 2014). In light of the increasing commercial and political interest in brain and biometric sensor applications, this trend is likely to continue.

With ground-breaking emerging technologies and the growing spread of Industry 5.0 and Society 5.0, AFFECT should be analyzed by taking into account demographic and cultural background, socioeconomic status, diversity attitudes, and context. Advanced computational models will be needed for this approach.

Quite a few biometric and neuroscience studies have been performed in the world, where AFFECT detection takes into account demographic and cultural background (age, gender, ethnicity, race, major diagnoses, and major medical history); socioeconomic status (education, income, and occupation); diversity attitudes; and context. Yet, to the best of our knowledge, none of the technologies available in the world offer AFFECT detection that incorporates political views, personality traits, gender, race, diversity attitudes, and cross-cultural differences in emotion.

Sometimes confusion exists in the spirit of some research about physiological effects due to emotional reactions and biometric patterns with regard to individual identification. To resolve this confusion, we analyze only physiological effects caused by emotional reactions (i.e., second generation biometrics; Section 3) in the part of the review discussing biometrics. Biometric patterns for individual identification are not analyzed in this research.

Human emotions can be determined by physiological signals, facial expressions, speech, and physical clues, such as posture and gestures. However, social masking—when people either consciously or unconsciously hide their true emotions—often renders the latter three ineffective. Physiological signals are therefore often a more accurate and objective gauge of emotions [529]. For instance, researchers [530,531] performed many studies to analyze physiological signals and unconscious emotion recognition. Nonetheless, our years of research experience have proven that in public spaces, facial expressions, speech, and physical clues, such as posture and gestures, are much more convenient and effective. 

Emotion recognition can be more accurate when human expressions are analyzed looking at multimodal sources such as texts, physiological signals, videos, or audio content [532]. Integrated information from signals such as gestures, body movements, speech, and facial expressions helps detect various emotion types [533]. Statistical methods, knowledge-based techniques, and hybrid approaches are three main emotion classification approaches in emotion recognition [534].

The emotional dimensions follow the approach of representing the emotion classes. Categorized emotions can be represented in a dimensional form with each emotion placed in a distinct position in space: either 2D (Circumplex model, “Consensual” Model of Emotion, Vector Model,) or 3D (Lövheim Cube, Pleasure-Arousal-Dominance [PAD] Emotional-State Model, Plutchik’s model, PAD Emotional-State Model), with each emotion occupying a distinct position in space. Most dimensional models have dimensions of valence and arousal or intensity or arousal dimensions: Valence dimension indicates how much and to what degree an emotion is pleasant or unpleasant, whereas arousal dimension differentiates between showing its state, either that of activation or deactivation [82]. The objectives of our study were most in line with Plutchik’s ‘wheel of emotions’ model, which we used in this research.

The use of artificial intelligence to recognize emotions and affective attitudes is a comparatively promising field of investigation. To make the most of artificial intelligence, multiple modalities in context should be generally used. Artificial intelligence has enabled biometric recognition and the efficient unpacking of human emotions and affective and physiological responses and has contributed considerably to advances in the field of pattern recognition in biometrics, emotions, and affective attitudes. Many different AI algorithms are used in the world, such as machine learning, artificial neural networks [535,536,537], search algorithms [166,538,539], expert systems [540,541], evolutionary computing [542,543], natural language processing [544,545], metaheuristics, fuzzy logic [546,547,548], genetic algorithm [549,550,551], and others. 

Based on our review, presented in Section 1, Section 2, Section 3, Section 4 and Section 5, we find that investigators should develop procedures to guarantee that AI models are appropriately used and that their specifications and results are reported consistently. There is a need to create innovative AI and machine learning techniques.

Based on the review (Section 1, Section 2, Section 3, Section 4 and Section 5), investigators should develop procedures to guarantee that AI models are appropriately used and that their specifications and results are reported consistently. There is a necessity to create innovative AI and machine learning techniques.

The existing emotion recognition approaches all need data, but the training of machine learning algorithms requires annotated data, and obtaining such data is usually a challenge [552]. The use of AI models may become less complex, and AI algorithms faster when certain database techniques are applied. These techniques can also provide AI capability inside databases. Supporting AI training inside databases is a challenging task. One of the challenges is to store a model in databases, so that its parallel training is possible with multiple tenants involved in its training and use, at the same that security and privacy issues are taken care of. Another challenge is to update a model, especially in case of dynamic data updates [553]. The following datasets can help with the task of classifying different emotion types from multimodal sources such as physiological signals, audio content, or videos: BED [554], MuSe [555], MELD [544,556], UIT-VSMEC [411] HUMAINE [557], IEMOCAP [558], Belfast database [559], SEMAINE [560], DEAP [561], eNTERFACE [384], and DREAMER [562]. Github [563], for instance, provides a list of all public EEG-datasets such as High-Gamma Dataset (128-electrode dataset from 14 healthy subjects with about 1000 four-second trials of executed movements, 13 runs per subject), Motor Movement/Imagery Dataset (2 baseline tasks, 64 electrodes, 109 volunteers), and Left/Right Hand MI (52 subjects).

The findings also suggest that the development of more powerful algorithms cannot address the perception, reading, and evaluation of the complexity of human emotions, by making an integrated analysis of users’ demographic and cultural background (age, gender, ethnicity, race, major diagnoses, and major medical history); socioeconomic status (education, income, and occupation); diversity attitudes; and context. We can only hope that the future will bring further research to address this issue and help to develop more advanced AFFECT technologies that can better cope with issues such as demographic and cultural background (age, gender, ethnicity, race, major diagnoses and major medical history); socioeconomic status (education, income and occupation); diversity attitudes; and context (weather conditions, pollution, etc.).

Worldwide research has yet to resolve several problems, and additional research areas have arisen, such as missing data analysis, potential bias reduction, a lack of stringent data collection and privacy laws, application of elicitation techniques in practice, open data and other data-related issues. Olivas et al. [564] for instance, analyze various methods for handling missing data:

- Missing data imputation techniques: analysis of the variable containing missing data (Mean, Regression, Hot Deck, Multiply Imputation) and analysis of relationships between variables for a case containing missing data (Imputation based on Machine Learning: Neural Network, Self-organizing map, K-NN, Multilayer perceptron);
- Case deletion (Listwise Deletion (Complete-case), Pairwise Deletion);
- Approaches that take into account data distributions (Bayesian methods, Model-based likelihood, Maximum Likelihood with EM).

**Figure 11:**
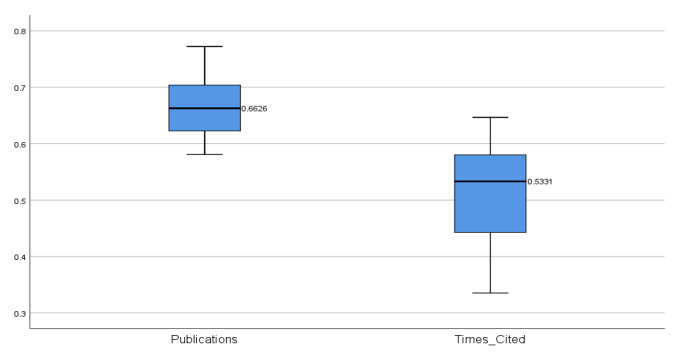
Distribution of correlations based on 15 criteria applied to 169 countries, their publications, and citations, as a CSP map.

It was found that the median correlation of the dependent variable of the Publications—Country Success model with the independent variables (0.6626) is higher than in the Times Cited—Country Success model (0.5331). Therefore, it can be concluded that the independent variables in the Publications—Country Success model are more closely related to the dependent variable than in the Times Cited—Country Success model (Figure 11).

**Figure 12:**
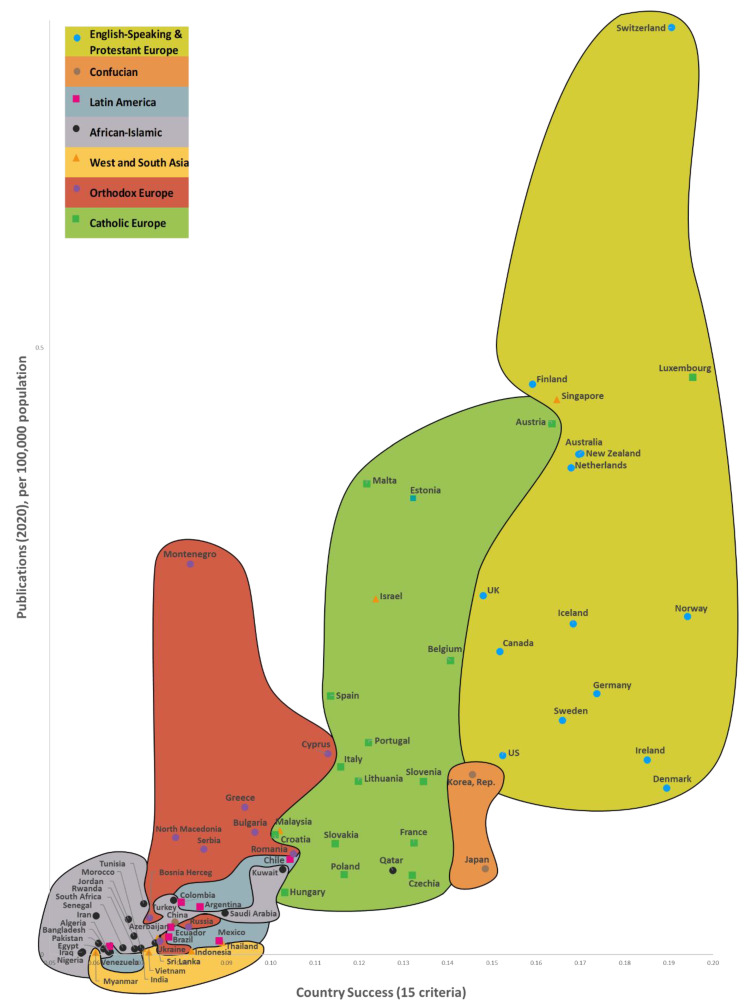
CSP map showing the success of countries in terms of the numbers of publications on AFFECT recognition (CSPN) in Web of Science journals with impact factor.

**Figure 13:**
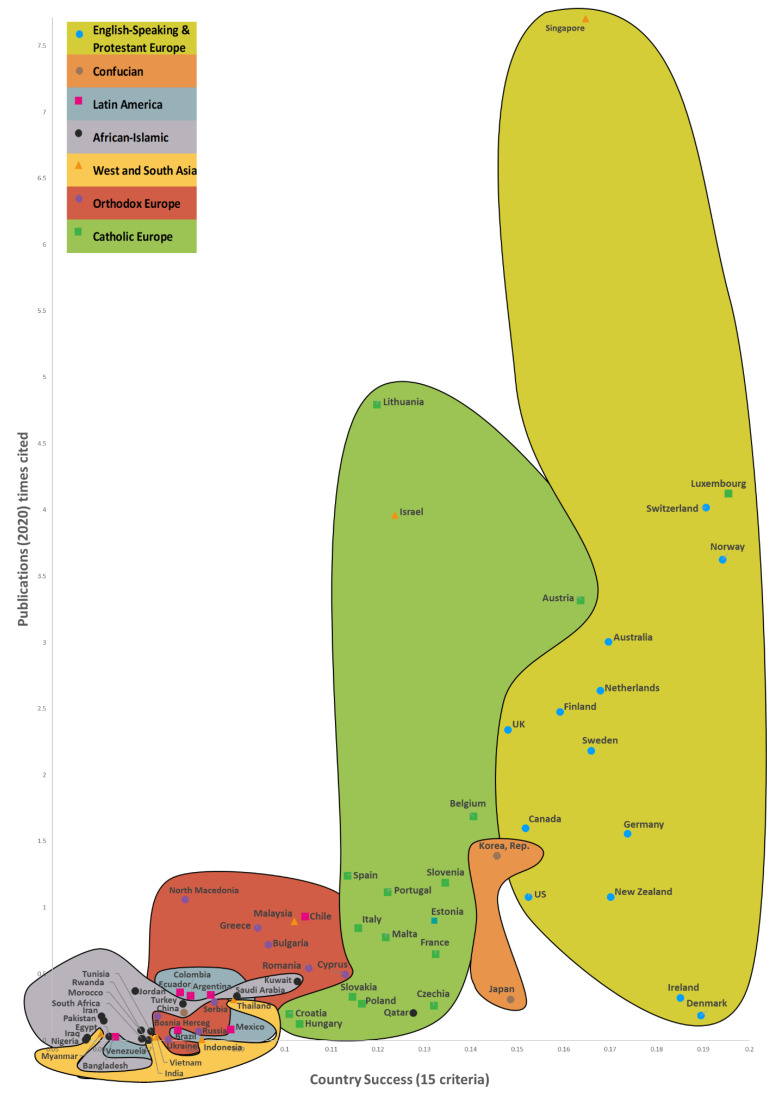
CSP map showing the success of countries in terms of the number of citations of their publications on AFFECT recognition (CSPC) in Web of Science journals with impact factor.

**Figure 14:**
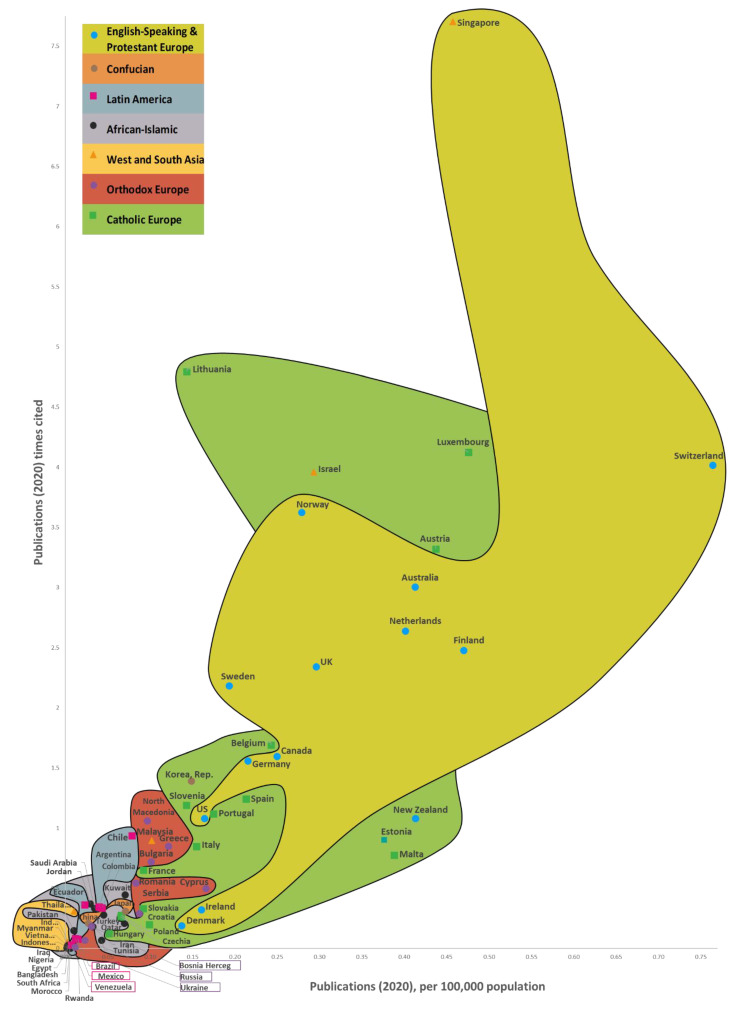
CSP map showing the number of articles on AFFECT recognition and the numbers of citations in Web of Science journals with impact factor.

The CSP maps of the world that have been compiled for this research provide a visualization of two aspects. A country’s success (x-axis) is one of the aspects, while the publications dimensions (CSPN and CSPC; y-axis) are the other (Figure 12 and Figure 13). The publications (x-axis) are one of the aspects, while the publications times cited dimensions (y-axis) are the other in Figure 14. The CSP maps group the countries into the same eight clusters as the Inglehart–Welzel 2020 Cultural Map of the World (English-speaking, Catholic Europe, Protestant Europe, Orthodox Europe, West and South Asia, African-Islamic, Confucian, and Latin America) [565]. Two clusters—English-speaking and Protestant Europe—have been merged into one because of their shared history, religion, cultures, and degree of economic development. The parallels between the two aforementioned clusters have been confirmed by numerous studies [566]. The Inglehart–Welzel 2020 Cultural Map of the World includes many institutional, technological, psychological, and economic variables that demonstrate strong perceptible correlations [567]. The country success indicators in the CSP maps can be characterized as a large set of variables within the criteria system, such as politics, human development and well-being, the environment, macroeconomics, quality of life, and values based.

In addition, this is a quantitative study to assess how the success of the 169 countries impacted the number of Web of Science articles published in 2020 on AFFECT recognition techniques that use brain and biometric sensors (or the latest figures available).

For the multiple linear regressions, we used IBM SPSS V.26 to build two regression models on 15 indicators of country success and the two predominant CSP dimensions. Two CSP regression models were developed based on an analysis of 15 independent variables and two dependent variables. The 15 independent variables and the two regression models are summarized in Table 4, Table 5, Table 6, Table 7 and Table 8. Table 4 contains descriptive statistics for two of the CSP models. The minimum and maximum values indicate the value range for each variable in the set of values that the variable in question can take. The average value of the full range that each variable can take is the mean and is usually equal to the arithmetical average. The standard deviation is a measure of the dispersion in the values of the variable in relation to the mean. Kurtosis is a measure of whether the values are heavy-tailed or light-tailed relative to the center of the distribution, whereas skewness is a measure of the symmetry of the distribution of the values. Acceptable values are considered to be between −3 and +3 for skewness, and between −10 and +10 for kurtosis. When the skewness is close to zero and kurtosis is close to three, the distribution of the values of the variable within the specified value range is in line with a normal distribution.

Step 9 entailed the construction of regression models for the number of publications and their citation rates, and the calculation of the ES indicators describing them. Two dependent variables and 15 independent variables were analyzed to construct these regression models. The process was as follows:

- Construction of regression models for the numbers of publications and their citations.
- Calculation of statistical effect size (ES) indicators describing these regression models. ES is a value used in statistics to measure the strength of the relationship between two variables, or to calculate a sample-size estimate of that amount [568]. An ES may reflect the regression coefficient in a regression, the correlation between two variables, the mean difference, or the risk of a specific event occurring [569]. Guidelines developed by Durlak [570] provide advice on the ESs to use in research, and how to calculate and interpret them. We used these guidelines, and applied the following five measures of ES, as these indicators are crucial for meta-analysis and could be computed from our measurements:
  ○ Pearson correlation coefficient (r): Beta weights and structure coefficients r are the two sets of coefficients that can provide a more perceptive stereoscopic view of the dynamics of the data [571]. Interpretation may be also improved through the use of other results (e.g., [572]).
  ○ Standardized beta coefficient (β): Theoretically, the highest-ranking variable is the one with the largest total effect, since β is a measure of the total effect of the predictor variables [573].
  ○ Coefficient of determination (R^2^): This is a measurement of the accuracy of a CSP model. The outcome is represented by the dependent variables of the model. The closer the coefficient of determination to one, the more variability the model explains. R^2^ can therefore be used to determine the proportion of the variation in the dependent variable that can be predicted by examining the independent variables [573].
  ○ Standard deviation: If this is too high, it will render the measurement virtually meaningless [574].
  ○ *p*-values. There is no direct relationship between the *p*-value and the size, and a small *p*-value may be associated with a small, medium, or large effect. There is also no direct relationship between the ES and its practical or clinical significance: a lower ES for one outcome may be more important than a higher ES for another outcome, depending on the circumstances [570].
- Calculation of non-statistical ES measures, which may better indicate the significance of the relationships between pairs of variables in our two models:
  ○ Research context: Durlak [570] argues that ESs must be interpreted in the context of other research.
  ○ Practical benefit: As this is an intuitive measure, practical benefit can allow stakeholders to make more accurate assessments of whether the research findings published can significantly improve their ongoing projects [575].
  ○ Indicators with low values: These are usually easier to improve than indicators with high values.

Based on the results of descriptive statistics, it can be concluded that the values of the dependent variables of the models used in the study demonstrate normal distribution (skewness < 10 and kurtosis < 10), which allows for the use of parametric analysis methods in the analysis.

A correlation analysis found that the strongest relationship in the Publications—Country Success model is between the dependent variable Publications and the independent variable GDP per Capita. Meanwhile, in the Times Cited—Country Success model, the strongest relationship is between the variables of Times Cited and GDP per Capita in PPP. It was also found that in both models, the relationships between the dependent variables and the independent variables are statistically significant (*p* < 0.001), except for the relationships between the dependent variables and the Unemployment Rate variable.

A reliability analysis of the compiled regression models allows us to conclude that the models are suitable for analysis (*p* < 0.05). It was also found that the changes in the values of the independent variables used in the models explain the variance of the Publications variable by 69.4%, and the variance of the Times Cited variable by 51.1%.

An analysis of the standardized coefficients of the model allows us to conclude that changes in the GDP per Capita variable have the biggest impact on changes in the Publications variable. The GDP per Capita in PPP variable also have a significant impact. Meanwhile, the Times Cited variable is most affected by the GDP per Capita in PPP variable, which has a statistically significant effect on the dependent variable.

To confirm Hypothesis 1, we built two CSP models, which are formal representations of the CSP maps. These models demonstrate that on average, an increase of 1% in a country’s success leads to an average improvement by 0.203% in the country’s two CSPN and CSPC dimensions. As the success of a country increased by 1%, the numbers of Web of Science articles published and their citations grew by 1.962% and 2.101%, respectively. Figure 12 and Figure 13 also illustrate that an increase in a country’s success goes hand in hand with a jump in its CSPN and CSPC dimensions, thus confirming Hypothesis 1. 

Hypothesis 2 was based on the results of the analysis pertinent to the CSP models, as well as on the correlations found between the 169 countries and the 15 indicators [66]. A clear visual confirmation of Hypotheses 1 and 2 are also provided by Figure 12 and Figure 13, which show the specific groupings of countries in the seven clusters examined in this study. These models may be of major significance for policy makers, R&D legislators, businesses, and communities.

## 7. Evaluation of Biometric Systems

In this chapter, we outline the rationale behind the current biometrics and brain approaches, compare the efficacy of existing methods, and determine whether or not they are capable of addressing the kinds of issues and challenges associated with the field (with figures). Biometric systems have several drawbacks in terms of their precision, acceptability, quality, and security. They are generally evaluated based on aspects such as (1) data quality; (2) usability; (3) security; (4) efficiency; (5) effectiveness; (6) user acceptance and satisfaction; (7) privacy; and (8) performance. 

Data quality measures the quality of biometric raw data [576,577]. This type of assessment is generally used to quantify biometric sensors and can also be used to enhance the system performance. According to the International Organization for Standardization ISO 13407:1999 [578], usability is defined as “[t]he extent to which a product can be used by specified users to achieve specified goals with effectiveness, efficiency, and satisfaction in a specified context of use” [579]:

- In this context, efficiency means that users must be able to accomplish the tasks easily and in a timely manner. It is generally measured as task time;
- Here, effectiveness means that users are able to complete the desired tasks without excessive effort. This is generally measured by common metrics such as the completion rate and number of errors, for example the failure-to-enroll rate (FTE) [580];
- User satisfaction measures the user’s acceptance of and satisfaction with the system. It is generally measured by looking at a number of characteristics, such as ease of use and trust in the system. Even if the performance of one biometric system exceeds that of another in terms of performance, this will not necessarily mean that it will be more operational or acceptable.

Security measures the robustness of a biometric system (including algorithms, architectures, and devices) against attack. The International Organization for Standardization ISO/IEC FCD 19792 [581] specifically addresses processes for evaluating the security of such systems [579]. 

Unlike traditional methods, biometric systems do not provide a 100% reliable answer, and it is almost impossible to obtain such a response. In a secure biometric system, there is a trade-off between recognition performance and protection performance (security and privacy). The reason behind this trade-off arises from the unclear concept of security, which requires a more standardized framework for evaluation purposes. If this gap can be closed, an algorithm could be developed that would jointly reduce both of them. ISO 19795 contained standards for performance metrics and evaluation methodologies for traditional biometric systems. In addition to performance testing, it provided metrics related to the storage and processing of biometric information [582]. ISO/IEC 24745 specifies that, unlike privacy, security is delivered at the system level. In general, the ability of a system to maintain the confidentiality of information with the use of the provided countermeasures (such as access control, integrity of biometric references, renewability, and revocability) is referred as its security factor. When seeking to bypass the security of a biometric system, an invader may impersonate a genuine user to gain access to and control over various services and sensitive data. Privacy refers to secrecy at the information level. The following criteria were proposed in ISO/IEC 24745 for the purpose of evaluating the privacy offered by biometric protection algorithms: irreversibility, unlinkability, and confidentiality [583]. 

The discriminating powers of all biometric technologies rely on the extent of entropy, with the following used as performance indicators for biometric systems [584,585,586,587]: False match rate (FMR); False non-match rate (FNMR); Relative operating characteristic or receiver operating characteristic (ROC); Crossover error rate or equal error rate (CER or EER); Failure to enroll rate (FER or FTE), and Failure to capture rate (FTC).

Specific advantages and disadvantages are characteristic to each biometric technology. Table 9 shows these comparisons.

Upon completing the literature analysis, we then compared biometric technologies looking at the following seven parameters: universality, distinctiveness/uniqueness, permanence, collectability, performance, acceptability, and circumvention (Table 10). Another set of comparisons was the strengths and weaknesses characteristic to biometric technologies and related to their ease of use, error incidence, accuracy, user acceptance, long term stability, cost, template sizes, security, social acceptability, popularity, speed, and whether or not they have been socially introduced (Table 11). The working characteristics of various biometrics differ, as does their accuracy, and depend on the design of their operation. The level of security and the kinds of possible errors are also different in each biometric approach; the denial of access to the biometric sample holders is possible caused by various factors such as aging, cold, weather conditions, physical damages, and so on [600,601]. Other researchers also look at FAR, FRR, CER, and FTE in their comparisons of biometric technologies (Table 12).

Multimodal biometric systems take advantage of multiple sensors or biometrics to remove the restrictions of unimodal biometric systems [616]. While unimodal biometric systems are restricted by the integrity of their identifier, the change of several unimodal systems having the same restrictions is low [617]. Multimodal biometric systems can fuse these unimodal systems sequentially, simultaneously, both ways, or in series, meaning sequential, parallel, hierarchical, and serial integration modes, respectively. For instance, final results of decision level fusion of multiple classifiers are joined using methods such as majority voting [616]. This multimodal analysis will assist in identifying the actual reasons of such issues with the current biometrics and brain approaches, as well as the restrictions of the existing state-of-the-art approaches and technologies.

An efficient way to combine multiple classifiers Is needed when an array of classifiers outputs is developed. Various architectures and schemes have been proposed for joining multiple classifiers. The most popular methods are majority vote and weighted majority vote. In majority vote, the right class is the one most selected by various classifiers. If all the classifiers show different classes or in the event of a tie, then the one with the highest overall output is chosen to be the right class. Vote averaging method averages the separate classifier outputs confidence for every class over the entire ensemble. The class output with the highest average value is selected to be the right class [618]. The vote averaging method has been used to measure the efficacy of existing biometrics methods (Table 10 and Table 11). In our case, High (Very High) was assigned 3 points, Medium was assigned 2, and Low was assigned 1. The calculations did not evaluate some qualitative indicators, such as error incidence and socially introduced. Additionally, not all biometrics technologies had data on the analyzed indicators. As a result, eye tracking we not evaluated in this case due to a lack of data. The highest average number of points was collected by Skin temperature-thermogram (2.57), Iris/pupil (2.43), Face (2.30), and Signature (2.09). Many of the metrics for biometric technologies in Table 9, Table 10, Table 11 and Table 12 are analyzed in detail throughout the article.

## 8. Discussion and Conclusions

Nevertheless, there are still unanswered questions that need to be addressed. We evaluated the evidence available to find a relationship between brain and biometric sensor data and AFFECT in order to determine the primary digital signals for AFFECT. The multidisciplinary literature used was from the disciplines of engineering, computer science, neuroscience, physiology, psychology, mathematical modeling, and cognitive science. The distinct conventions of these disciplines resulted in certain variegations, depending on the features and characteristics of the research results being focused on. The literature under analysis has small sample sizes, short follow-up times, and significant differences in the quality of the reports, which limits the interpretability of the pooled results. On average, the current AFFECT detection techniques that use brain and biometric sensors achieved a classification accuracy greater than 70%, which seems sufficient for practical applications. As part of this review, several issues that need to be addressed were identified, as well as numerous recommendations and directions for future AFFECT detection and recognition research being suggested. They are listed below:

- Many studies fail to report information on demographic and cultural background, socioeconomic status, diversity attitudes, and context, and AFFECT papers often have limited descriptions of feature extraction and analysis. This has a significant impact on the interpretation of their findings. Sample recommendations include reporting on participant enrolment and selection approaches and analysis of demographic and cultural background (age, gender, ethnicity, race, major diagnoses, and major medical history); socioeconomic status (education, income and occupation), diversity attitudes, and context. In order to improve the ability of researchers to assess the strength of evidence, one of the first steps should be the development of this kind of consistent reporting.
- Behavioral traits (e.g., gesture, keystroke, voice) change over time, and therefore are less stable. Multiple interactions are typically required to set a reliable baseline. Injury, illness, age, and stress can also cause changes in behavioral traits. Many of the studies on AFFECT recognition examined brain and biometric data under different AFFECT while overlooking the baseline (spontaneous) brain and biometric data.
- The literature did not contain brain and biometric sensor-based AFFECT recognition of mixed emotions (parallel involvement of negative and positive emotions). We study the 30 primary, secondary, and tertiary dyads of Plutchik’s wheel of emotions, creating mixed emotions.
- Researchers need a set of guidelines to ensure AI models (artificial neural networks, evolutionary computing, natural language processing; metaheuristics, fuzzy logic, genetic algorithm) are correctly applied, and that their specifications and results are consistently reported (the model selection strategy, parameter estimates in the model with confidence intervals, performance metrics, etc.). There is also a need to further develop advanced AI and machine learning techniques (multi-modal learning, neuroscience-based deep learning, automated machine learning, self-supervised deep learning, Quantum ML, Tiny ML, System 2 deep learning).
- More results are also needed to identify which of the elicitation techniques applied in practice are effective, and in which cases they work best, taking into account the type of information obtained, the stakeholders’ (developers, end-users, etc.) characteristics, the context, and other factors. More data sets need to be created that use active elicitation techniques, such as various games, as these are better at mimicking real-life experiences and bringing about emotions. Gamification is a current trend that uses game methods for real-life AFFECT elicitation.
- Recommendations also state that the two sources of potential bias (AFFECT interpretation algorithmic biases, data sources and input) in multi-feature studies should be reduced, and a wider variety of multimodal samples should be used.
- Missing data analysis has some gaps, for example missing data descriptions and how missing data is handled, and most appropriate methods should be applied in AFFECT recognition. As far as missing data goes, the literature had major shortcomings.
- As algorithms improve, accuracy is growing, but this significantly depends on the data sets used. Some gaps and a lack of discussion have also been noted concerning the question of whether the integrated brain and biometric sensors used in this research are reliable and appropriate for AFFECT detection.
- A trend related to emotional AI businesses (Realeyes, Affectiva, etc.) that expand their global operations in regions with less stringent data collection and privacy laws has not been sufficiently examined globally.
- The recommendations for open science include the proposal to share and reuse open multimodal AFFECT data, information, knowledge, and science practices (publications and software) by preparing a Data Management Plan that would address any important aspects of making data findable, accessible, interoperable, and reusable, or FAIR. Open data analysis should also include recognized and validated scales for AFFECT evaluation; any accessible confirmation on the reliability and validity of the AFFECT device and sensor applied should be presented. The open datasets have usually sought to obtain higher accuracy by using different sets of stimuli and groups of participants.

Emotional acculturation, happens when people, on contact with a different culture, learn new ways to express their emotions [619], incorporate new cultural values in their existing set, and then adjust their emotions to suit these new values [620,621,622,623]. This may be a research area in affective computing that needs more studies and focus. With growing global integration, emotional acculturation will become increasingly important, and advanced computational models will be needed to simulate the related processes. M.-T. Ho et al. [624] believe that this may be a key thematic change in the decades to come. The findings also suggest that developing more powerful algorithms cannot solve the perception, reading and evaluation of the complexity of human emotions. Instead, the complex modulators that affective and emotional states stem from need to be better understood by the scientific community. We can only hope that the future will bring further research that will remedy this and help develop more advanced technologies that can better cope with issues such as gender, race, diversity attitudes, and cross-cultural differences in emotion [624].

The substantial improvements in the development of affordable and simple to utilize sensors for recognizing AFFECT have resulted in numerous studies being conducted. For this review, we studied in detail 634 articles. We focused on recent state-of-the-art AFFECT detection techniques. We also took existing data sets into account. As this review illustrates, exploring the relationship between brain and biometric signals and AFFECT is a formidable undertaking, and novel approaches and implementations are continually being expanded.

The evaluation of the intensity of human AFFECT is a complex process which requires the use of a multidirectional approach. The main difficulties of this process include variations in the nature of human beings, social aspects, etc., due to these methods, which fits for average evaluation of customers majority, but shows poor results in personalized cases and vice versa. Moreover, the reliability of evaluations of human emotions strongly depends on the number of biometric parameters used, and the measurement methods and sensors applied. It is well known that a higher reliability of recognition can be achieved by increasing the number of parameters, but this will also increase the need for certain equipment and will slow down the evaluation process. The selection of measurement methods and sensors is no less important in the successful recognition of emotions. Contact measurement methods give the most reliable results, but their implementation is relatively complicated and may even be frightening for potential customers. The best solution in this case is non-contact measurement methods, that is, contact methods which do not require special preparation and allow measurements to be taken without the knowledge of the customer.

Future research possibly could focus on areas of reaction to emotion development stage, while sensing and evaluation became faster than emotion recognition by person itself.

This research has addressed the various issues that emerge when affective and physiological states, as well as emotions, are determined by recognition methods and sensors and when such studies are later applied in practice. The manuscript presents the key results on the contribution of this research to the big picture. These results are summarized below:

- Many studies around the world apply neuroscience and biometric methods to identify and analyze human valence, arousal, emotional and physiological states, and affective attitudes (AFFECT). An integrated review of these studies is, however, yet missing.
- In view of the fact that no reviews of AFFECT recognition, classification and analysis based on Plutchik’s wheel of emotions theory are available, our study has examined the full spectrum of thirty affective states and emotions defined in the theory.
- We have demonstrated the identification and integration of contextual (pollution, weather conditions, economic, social, environmental, and cultural heritage) [342] and macro-environmental [568] data with data on AFFECT states.
- The authors of the article have presented their own Real-time Vilnius Happiness Index (Figure 10a) and other systems and outputs to demonstrate several of the aforementioned new research areas in practice.

Information on diversity attitudes, socioeconomic status, demographic and cultural background, and context is missing in many studies. In this study, we have identified real-time context [347] data and have integrated them with AFFECT data. For example, the ROCK Video Neuroanalytics system and associated e-infrastructure were established as part of the H2020 ROCK project, in which passers-by were tracked at 10 locations across Vilnius [348]. One of the outputs was the real-time Vilnius Happiness Index (Figure 10 and https://api.vilnius.lt/happiness-index, accessed on 5 September 2022), and the project also involved a number of additional activities (https://Vilnius.lt/en/category/rock-project/, accessed on 5 September 2022) [625,626].

The analysis of the global gap In the area of affective biometric and brain sensors presented in this study and our aim of contributing to the current state of research in this area have led to the aforementioned research results.

Based on the evaluation of biometric systems performed in Section 7 and the conclusions presented in Chapter 8, future AFFECT biometrics and neuroscience development directions and guidelines are visible. We performed the above analysis by extensively discussing biometric and neuroscience methods and domains in the article.

Additionally, Section 2 and Section 6 present statistical and multiple criteria analysis across 169 nations, our outcomes demonstrate a connection between a nation’s success, its number of Web of Science articles published, and its frequency of citation on AFFECT recognition. This analysis demonstrates which country’s success metrics significantly influence future AFFECT biometrics and neuroscience development.

Advancements in the development of biometric and neuroscience sensors and their applications are summarized in this review. Regardless of the encouraging progress and new applications, the lack of replicated work and the widely divergent methodological approaches suggest the need for further research. The interpretation of current research directions, the technical challenges of integrated neuroscience and affective biometric sensors, and recommendations for future works are discussed. The reviewed literature revealed a host of traditional and recent challenges in the field, which were examined in this article and are presented below.

Biometric research aims to provide computers with advanced intelligence so that they can automatically detect, capture, process, analyze, and identify digital biometric signals—in other words, so they can “see and hear”. In addition to being one of the basic functions of machine intelligence, this is also one of the most significant challenges that we face in theoretical and applied research [627]. 

There are still many challenging issues in terms of improving the accuracy, efficiency, and usability of EEG-based biometric systems. There are also problems concerning the design, development and deployment of new security-related BCI applications, such as personal authentication for mobile devices, augmented and virtual reality, headsets and the Internet [628]. Albuquerque et al. [628] have presented the recent advances of EEG-based biometrics and addressed the challenges in developing EEG-based biometry systems for various practical applications. They have also put forth new ideas and directions for future development, such as signal processing and machine learning techniques; data multimodal (EEG, EMG, ECG, and other biosignals) biometrics; pattern recognition techniques; preprocessing, feature extraction, recognition and matching; protocols, standards and interfaces; cancellable EEG biometrics; security and privacy; and information fusion for biometrics involving EEG data, virtual environment applications, stimuli sets and passive BCI technology.

Some of these challenges (accuracy, efficiency, usability, etc.) are analyzed in the article. Each of these features can be examined in more detail. For example, Fierrez et al. [629] analyzed five challenges in multiple classifiers in biometrics: design of robust algorithms from uncooperative users in unconstrained and varying scenarios; better understanding about the nature of biometrics; understanding and improving the security; integration with end applications; understanding and improving the usability. “Design of robust algorithms from uncooperative users in unconstrained and varying scenarios” is a challenge that has been a major focus of biometrics research for the past 50 years [2], but the performance level for many biometric applications in realistic scenarios is still not adequate [629].

Recently, new challenges in the field have been appearing; some of which are presented below as an example. Sivaraman [630] argues that in the age of AI and machine learning, cyberattacks are more powerful and are sometimes able to crack biometric systems. Additionally, these attacks will become more frequent. Multimodal biometrics are increasingly important, where a combination of biometrics is used for greater security. The pandemic has resulted in changes to the biometric algorithm of various modalities. Facial recognition algorithms have been improved to recognize people wearing masks and cosmetics. Updates like these may improve the accuracy of biometrics systems. Biometric devices will take web and cloud-based applications to the next level, as many organizations will continue to operate remotely [630].

Furthermore, a few problems have not been solved, and additional research fields have emerged, namely: biometric and neuroscience technologies lack privacy, are invasive and persons do not like to share their personal data and be identified; lack of protection from hacking; lack of accuracy; a quite expensive life cycle (brief, design, development, set up, running, operation, etc.); lack of capability to read some human features; customer satisfaction is not always guaranteed; human figure form recognition and examination of figure fragments, examination of head vibrations, and human electrical fields are inefficient.

## Figures and Tables

**Table 1:** Traditional non-invasive neuroscience methods.

Methods	Author(s)	Description
Electroencephalography (EEG)	[111,253,254,255,256,257,258,259,260,261,262,263,264,265,266]	EEGs capture brainwave variations, using recorded amplitudes to monitor mental states that include alpha waves (relaxation), beta waves (wakefulness), delta waves (sleep), and theta waves (calmness) [255]. An EEG signal comprises five brain waves and measuring the activity of certain brain areas can reveal the state of the subject’s cortical activation. Each wave is characterized by different amplitudes and frequencies, and corresponds to distinct cognitive states [265].
Magnetoencephalography (MEG)	[111,253,254,255,256,259,260,267]	Using magnetic potentials, an MEG records brain activity at the scalp level. A helmet with sensitive detectors is placed on the subject’s head to track the signal [255], and the MEG detects the magnetic fields produced by electromagnetic fields [111].
Transcranial Magnetic Stimulation (TMS) (Figure 3)	[111,251,253,255,258,260,267]	TMS modulates the activity of certain brain areas located 1–2 cm below the skull, without reaching the neocortex, using magnetic induction [255]. When TMS is used, short electromagnetic impulses are applied at the scalp level. This instrument can stimulate or inhibit a particular cortical area [111].
Near Infrared Spectroscopy (NIRS)	[267,268,269]	NIRS measures hemodynamic alterations accompanying brain activation and is a simple bedside technique [269]. NIRS makes use of the near-infrared region of the electromagnetic spectrum (about 700–2500 nm). Measurements are taken of light scattered from the surface of and through a sample, and NIR reflectance spectra can give rapid insight into the properties of a material without altering the sample [268].
Steady-State Topography (SST)	[251,253,255,256,260]	SST can be applied to track high-speed changes and measure the activity of the human brain. This tool is very commonly used in neuromarketing research and cognitive neuroscience [255].
Functional Magnetic Resonance Imaging (fMRI) (Figure 4)	[111,251,253,254,255,256,258,259,260,261,263,264,266,267]	fMRI is suitable for use within neuromarketing studies, as brain activity can be measured in subjects performing certain tasks or experiencing marketing stimuli. It allows for the observation of deep brain structures, and hence can reveal patterns [255]. fMRI can also measure increases in oxygen levels in the blood flow to the brain and can detect the active cortical regions [111].
Positron Emission Tomography (PET) (Figure 4)	[111,251,253,254,256,259,260,261,267]	The subject is injected with a radioactive substance, and the flow of the substance is then measured. Significant increases in the flow are seen in activated areas [111].
Diffusion Tensor Imaging (DTI) (Figure 5)	[267,270,271]	This is an MRI-based neuroimaging technique that allows the user to estimate the location, anisotropy and orientation of the brain’s white matter tracts [271]. DTI makes it possible to visualize and characterize white matter fasciculi in two and three dimensions [270].

**Table 2:** Physiological and behavioral biometrics.

Technique	Author(s)	Description
Physical/Physiological Features		
Eye Tracking (ET) (Figure 6)	[111,251,253,254,255,256,257,258,259,260,261,264,265,266,267]	ET determines the areas at which the subject is looking and for how long, and also tracks the movement of the subject’s eyes and changes in pupil dilation while the subject looks at stimuli. With this technique, behavior and cognition can be studied without measuring brain activity [255]. By measuring eye movements and visual attention, an eye tracker determines the point of regard [265].
Blinking	[261,264,293]	Eye blinking forms the basis of the new biometric emotions identifier proposed by Abo-Zahhad et al. [293]. These authors outline where eye blinking signals come from and give an overview of the features of the EOG signals from which the eye blinking waveform is extracted.
Iris characteristics		User-oriented examinations were applied to find the relationships between personality and three common iris characteristics: pigment dots, crypts, and contraction furrows [294]. Dark-eyed individuals typically have higher scores for neuroticism and extraversion [295], sociability [296], and ease of emotional arousal [297].
Facial Action Coding (FC)/Facial Expression Analysis Surveys (Figure 7)	[253,254,255,256,257,258,260,261,263,264,265,298]	FC uses a video camera to track micro-expressions that correspond to certain subconscious reactions. The activity of the facial muscles is tracked [255]. Scientists and practitioners have developed various open data datasets (KaoKore Dataset, CelebFaces At-tributes Dataset, etc.) and applied elicitation techniques (gamification, virtual reality) in practice.
Facial Electromyography (fEMG) (Figure 8)	[251,253,254,255,256,259,260,261,262,263,298,299]	fEMG is used in measuring and evaluating the physiological properties of facial muscles [255].
Odor	[300]	This a method of emotion recognition based on an individual’s odor [300]. An emotional mood, for example a period of depression, may affect body odor [301].
Keystroke dynamics and mouse movements (Figure 9)	[302]	AFFECT states can be determined by how a person moves a computer mouse while sitting at a computer.
Skin Conductance (SC)/Galvanometer or Galvanic Skin Response (GSR)	[111,251,253,255,256,258,260,261,262,264,265,267]	SC is highly correlated with the rate of perspiration, and is often linked to stress as well as to the processes happening in the nervous system [261]. SC methods measure arousal based on tiny changes in conductance that occur when something activates the autonomic nervous system [255]. The sympathetic branch of the autonomic nervous system controls the skin’s sweat glands, and the activity of the glands determines the galvanic skin response [265].
Heart rate (HR)/Electrocardiogram (ECG)	[19,111,251,256,261,303]	An ECG is used to measure the electrical activity of the heart [261]. An ECG relies on cardiac electrical activity and measures the electrical impulses that travel through the heart with each beat, causing the heart muscle to pump blood. In ECGs of a normal heartbeat, the timing of the lower and top chambers of the heart is charted [303].
Respiratory Rate Assessment (RRA)	[111,261,304]	Respiratory rate, one of fundamental vital signs, is sensitive to various pathological situations (clinical deterioration, pneumonia, adverse cardiac events, etc.), as well as stressors [304].
Skin temperature (SKT)	[305]	SKT data can be used to measure the thermal responses of human skin. SKT depends on the complex relationship between blood perfusion in the skin layers, heat exchange with the environment, and the central warmer regions of the skin [305]
Photoplethysmography (PPG) or Blood volume pulse (BVP)	[305]	Changes in the amplitudes of PPG signals are related to the level of tension in a human being. PPG is a simple, non-invasive method of taking measurements of the cardiac synchronous changes in the blood volume [305].
Trapezium electromyogram	[306]	EMG is a technique that can be used to evaluate and record the electrical activity generated by skeletal muscle [306], for example the trapezius muscle [307].
Neurotransmitter (NT)	[251,308]	Brain neurotransmitters are particular chemical substances that act as messengers in chemical synaptic transmissions and can transmit emotive information. They have excitability and inhibitive abilities [308].
Voice/Speech/Voice Pitch Analysis (VPA)	[263,267,300,309,310]	This is a method of emotion recognition that relies on the person’s voice.
Implicit Association Test (IAT)	[255,264,311]	IAT measures individual behavior and experience by assessing the reaction times of subjects to determine their inner attitudes. The subjects are given two cognitive tasks, and measurements are taken of the speed at which they associate two distinct concepts (brands, advertisements, etc.) with two distinct assessed features. IATs can be used to identify hierarchies of products by means of comparisons [255].
Mouse Tracking (MT)	[257,312]	Recognition of a user’s emotions is possible based on their mouse movements. Users can be classified by extracting features from raw data on mouse movements and employing complex machine learning techniques (e.g., a support vector machine (SVM)) and basic machine learning techniques (e.g., k-nearest neighbor) [312].
Signature (Figure 9)	[298,299,300,309]	Emotions can be identified by their handwriting style, and in particular their signature.
Gait (Figure 9)	[298,299,300,309]	This method allows for emotions recognition based on a person’s walking style or gait [300].
Lip Movement	[299]	Lip movement measurements are a recently developed form of biometric emotions recognition that is very similar to the way a deaf person determines what is being said by tracking lip movements [299].
Gesture	[298,309]	Gesture recognition is used to identify emotions rather than a person, and gestures are grouped into certain categories [298].
Keystroke/Typing Recognition (Figure 9)	[169,300]	In this method, the unique characteristics of a person’s typing style are used for emotions identification purposes [300].

**Table 3:** An overview of studies on arousal, valence, affective attitudes, and emotional and physiological states (AFFECT) recognition.

Stimulus	AFFECT	Methods	Reference
Recording of dances, video	Anger, fear, grief, and joy	GSR, eye movement (Figure 6)	[334]
Neurophysiological research from 2009 to 2016	Overview of the existing works in emotion	EEG	[335]
Affective stimuli	Surprise, disgust, anger, fear, happiness, and sadness	EEG	[336]
The visual stimuli, black and white photographs of 10 different models	Happy, sad	MEG	[337]
20 face actors, each displaying happy, neutral, and fearful facial expressions	Happy, neutral, fearful	MEG	[338]
Task-irrelevant emotional and neutral pictures	Pleasant, unpleasant	TMS	[339]
A subset of music videos from the Dataset for Emotions Analysis using Physiological signals (DEAP) dataset	Valence, arousal	fNIRS, EEG	[340]
Emotional faces for the emotion perception test	Pleasant, unpleasant, neutral	fMRI	[341]
-	Stress	PET	[342]
Video	Happiness, sadness, disgust, anxiety, pleasant, unpleasant, neutral	PET	[343]
Facial Emotion Selection Test (FEST)	Positive, negative	DTI	[344]
Real time biometric-emotional data collection from depersonalized passersby	Neutral, happiness, sadness, surprised, anger, scared, valence, arousal, disgust, interest, confusion, boredom	Emotional, Affective and Biometrical States Analytics of the Built Environment Method	[345]
Real time data collection	Happy, sad, angry, surprised, scared, disgusted, valence, arousal	Method of an Affective Analytics of Demonstration Sites	[346]
Scanning a human-centered built environment, real time data collection	Sadness, disgust Happiness, anger, fear surprise, boredom, neutral, arousal, valence, confusion, and interest	Affect-Based Built Environment Video Analytics	[347]
Remote real time data	Happiness, arousal, valence	Video Neuro-advertising Method	[93,348]
Smelling strips	Happy, radiant, well-being, soothed, energized, romantic, sophisticated, sensual, adventurous, comforted, amused, interested, nostalgic, revitalized, self-confident, surprised, free, desirable, daring, excited	IRT	[349]
Text	Positive and negative valence	Eye tracking (ET)	[350]
21 video fragments	High/low arousal, high/moderate/low valence	Eye tracking (ET)	[351]
Crypts	Feelings, tendermindedness, warmth, trust and positive emotions	Iris	[294]
The simulation environment	Wellness/malaise, relaxation/tension, fatigue/excitement	Retina	[352]
Colors	Surprise, Happiness, Disgust, Anger, Sadness and Fear	Blinking, heart rate	[353]
HSV color space	Fear, disgust, surprise, joy, anticipation, sadness, anger, trust	Blinking	[354]
Review of existing novel facial expression recognition systems	Anger, disgust, fear, happiness, sadness, surprise and neutral	Facial expression recognition	[355]
Destination promotional videos	Pleasure, arousal	Skin conductance, facial electromyography	[355]
Games scenario between a human user and a 3D humanoid agent	Arousal, valence, fear, frustrated, relaxed, joyful, excited	Electromyography, skin conductance	[356]
Dramatic film	Real-time emotion estimation	EEG, Heart Rate, Galvanic Skin Response	[357]
Emotional state of a driver while in an automobile	Happy, anger	Electrocardiogram (ECG)	[358]
Music	Pleasure, unpleasure	Heart and respiratory rates	[359]
Trier Social Stress Test	Stress, relax	Respiratory rate and heart rate	[360]
Voice- and speech-pattern analysis	Normal, angry, panic	Voice, speech	[361]
Implicit anxiety-related self-concept	Shame, guilt proneness, anxiety, anger-hostility	Implicit Association Test	[362]
Case studies	Self-control, happiness, anger, fear, sadness, surprise, and anxiety	Mouse Tracking	[302]
Academic study website	Neutral, positive, negative	Mouse Tracking	[363]
Motor improvisation task	Joy, sadness, and a neutral control emotion	Signature	[364]
-	Neutral, joy, anger, sadness	Gait	[365]
Text	Neutral, joy, surprise, fear, anger, disgust, sadness	Lip Movement	[366]
Dataset	Anger, disgust, fear, happiness, sadness, and surprise	Keystroke dynamics	[367]
Recall of past emotional life episodes	Valence, arousal	EEG	[368]
Physiological emotional database for real participants	Valence, arousal	Peripheral signals, EEG	[369]
Data from wearable sensors on subject’s skin	High/neutral/low arousal and valence	ECG, EEG, electromyography (EMG)	[370]
Real time heartbeat rate and skin conductance	High/low arousal and valence	GSR, temperature, breathing rate, blood pressure, EEG	[371]
Multimedia contents based on IPTV, mobile social network service, and blog service	Pleasant, unpleasant	GSR, skin temperature, heart rate	[372]
Stress stimuli	High/low valence, high/low arousal	GSR, heart rate, ECG	[373]
CCD-capture human face, measure user’s physiological data	Pleasant, unpleasant	GSR, photoplethysmogram (PPG), skin temperature	[374]
Music videos	High/low arousal, high/low valence	EEG	[375]
Detect the current mood of subjects	High/low arousal, high/low valence	EEG	[376]
DEAP database	Joy, fear, sadness, relaxation	EEG, back-propagation neural network	[377]
Hjorth features, statistics features, high order crossing features	Happy, calm, sad, scared	EEG, CNN, LSTM recurrent neural networks	[378]
Thirty film clips	Serenity, hope, joy, awe, love, gratitude, amusement, interest, pride, inspiration	EEG	[379]
Transcendental meditation	Ecstasy	EEG	[380]
Ultimatum game	Acceptance	EEG	[381]
Driving a car equipped	Trust	EEG, GSR	[382]
12 prototypes that were designed based on the framework of diachronic opposite emotions	Amazement, happiness	EEG, SD tests	[383]
Audio-visual emotion database	Pleasure, irritation, sorrow, amazement, disgust, and panic	-	[384]
Sleep measures	Grief	EEG	[385]
Real episodes from subjects’ lives	Grief, anger	EEG	[386]
Virtual environment consisting of three types of cues	Pensiveness relaxation, non-arousal, stress	EEG	[387]
Patient with dramatic, episodic, seizure-related rage and violence	Rage and aggression	Video-EEG recording	[388]
DEAP database	Rage	EEG, multiclass-common spatial patterns	[389]
Brainstem auditory evoked potentials	Rage and self-injurious behavior	EEG, brainstem evoked potentials (BAEPs)	[390]
Acoustic annoyance	Annoyance	EEG	[391]
70 dBA white noise and pure tones at 160 Hz, 500 Hz and 4000 Hz	Annoyance	EEG	[392]
30 pictures from International Affective Picture System	Neutral, joy, sadness anger, surprise, valence (positive and negative), contempt, fear, disgust	EEG	[393]
Movie clips	Anger, fear, anxiety, disgust, contempt, joy, happiness	EEG	[394]
Emotional factor	Aggressiveness	EEG	[395]
Buss–Durkee questionnaire	Aggressiveness	EEG	[396,397]
Reward anticipation	Anticipation	EEG	[398]
Structured Clinical Interview for DSM-IV	Anticipation	EEG, fMRI	[399]
DEAP database	High/low valence and arousal	EEG	[400,401,402,403,404,405]
Reading and reflection task about Muslims	Disapproval	EEG, ANOVA	[406]
Simulated train driving	Fatigue and distraction	EEG, Multi-type feature extraction, CatB-FS algorithm	[407]
Faces (the participant’s own face, the face of a stranger, and a celebrity’s face)	Admiration	EEG, 18-Items Narcissistic Admiration and Rivalry Questionnaire	[408]
Presentation of 12 virtual agents	Acceptance	EEG and the virtual agent’s acceptance questionnaire (VAAQ)	[409]
English prosocial and opposite antisocial words in a sentence	Approval and disapproval	EEG, ANOVA	[410]
Data from Facebook comments	Enjoyment (peace and ecstasy), sadness (disappointment and despair), fear (anxiety and terror), anger (annoyance and fury), disgust (dislike and loathing) surprise, other (neutral)	Natural language processing (NLP); convolutional neural network (CNN) and long short-term memory (LSTM); Random Forest and support vector machine (SVM), standard Vietnamese social media emotion corpus (UIT-VSMEC)	[411]
Video clips	Pride, love, amusement, joy, inspiration, gratitude, awe, serenity, interest, hope	fNIRS	[412]
User’s interaction with a web page	Arousal/valence anxiety and aggressiveness	Facial expressions, Facial Action Coding System, specialized questionnaires	[413]
An investment game that uses artificial agents	Trust	EEG	[285]
Simulated autonomous system	Trust	EEG and GSR	[382]
The iCV-MEFED dataset. For each subject in the iCV-MEFED dataset, five sample images were captured.	Neutral, angry, contempt, happy, happily surprised, surprisingly fearful, surprised	Facial emotion recognition (Figure 7), CNN; Inception-V3 network	[414]
Dynamic emotional facial expressions were generated by using FACSGen	Contempt, disgust, sadness, neutral	ANOVA, Participants completed emotion scales	[415]
Film clips	Pride, love, amusement, joy, inspiration, gratitude, awe, serenity, interest, hope	EEG, multidimensional scaling (MDS), intra-class correlation coefficients (ICCs)	[379]
Simulated driving system	Vigilance	EEG and forehead electrooculogram (EOG), eye tracking (Figure 6)	[416]
DEAP dataset	Optimism, pessimism, calm	EEG, CNN	[166]
Music	Relaxing-calm, sad-lonely, amazed-surprised, quiet-still, angry-fearful, happy-pleased	Binary relevance (BR), label powerset (LP), random k-label sets (RAKEL), SVM	[417]
Music	Happiness, love, anger and sadness	EEG, SVM, Multi-Layer Perceptron (MLP), and K-nearest Neighbor (K-NN)	[418]
Three sets of pictures	Anticipation	Facial emotions (Figure 7), action observation network (AON), two-alternative forced-choice procedure, Reaction times (RT), ANOVA	[419]
Individuals enacted aggressive actions, angry facial expressions and other non-aggressive emotional gestures	Aggressive actions and anger	Kinect infrared sensor camera: hand movement, body posture, head gesture, face (Figure 9), and speech. SVM and the rule-based features	[420]
Images of faces from the Ekman and Friesen series of Pictures of Facial Affect	Grief	Facial Expression of Emotion Test (Figure 7)	[421]
Music	Soothing, engaging, annoying and boring	FBS fusion of three-channel forehead biosignals, ECG	[422]
Films	Amusement, anger, grief, and fear	Fingertip blood oxygen saturation (OXY), GSR, HR	[423]
Polish emotional database, database consists of 12 emotional states	Rage, anger, annoyance, grief, sadness, pensiveness, ecstasy, joy, serenity, terror, fear, apprehension	Speech, KNN Algorithm	[424]
Video	Nonverbal behaviors signaling dominance and submissiveness	Implicit association test, body language, MANOVA	[425]
Music	High/low valence, high/low arousal	EMG, EEG, HRV, GSR	[426]
The external auditory canal is warmed or cooled with water or air	High and low arousal	Electrodermal activity (EDA), HRV, activity tracker, EMG, SKT	[427]
After-image experiments, direct visual observation, photography of the eyes, recording of the corneal reflex	High/low valence, high/low arousal	GSR, EMG	[428]
Assessment of emotional states experienced by racing drivers	Sadness, fear, anger, surprise, happiness, and disgust	ECG, EMG, respiratory rate, GSR	[429]
Dataset of standardized facial expressions	Happiness, sadness, anger, disgust, fear, and surprise	Facial Action Coding (FC)	[430]
Neighbor sounds	Arousal, valence	fEMG, heart rate (HR), electrodermal activity (EDA)	[431]
Audio visual stimuli	Joy, sadness, anger, fear	ECG	[432]
Playing with the infant to elicit laughter	Joy	Skin temperature (SKT)	[433]
Two different kinds of video inducing happiness and sadness	Happiness, sadness	Photoplethysmography (PPG), skin temperature (SKT)	[434]
International Affecting Picture System (IAPS) pictures	Joy, sadness, fear, disgust, neutrality, amusement	Electromyogram signal (EMG), respiratory volume (RV), skin temperature (SKT), skin conductance (SKC), blood volume pulse (BVP), heart rate (HR)	[435]
Movie and music video clips	Arousal, valence	Electrooculogram (EOG), electrocardiogram (EEG) trapezium electromyogram (EMG)	[436]
Audio/visual	Anger, happiness, sadness, pleasure	GSR, EMG, respiratory rate, ECG	[437]

**Table 4:** Descriptive statistics for the dependent variables of two models.

Descriptive Statistics	Descriptive Statistics of 2 Models Dependent Variables	
	Publications—Country Success	Times Cited—Country Success
	Model 1 (CSPN)	Model 2 (CSPC)
Mean	0.1354	0.9279
Median	0.0785	0.3297
Maximum	0.7642	7.7034
Minimum	0.0015	0.0000
Standard Deviation	0.1557	1.3893
Skewness	1.5533	2.4316
Kurtosis	5.3614	9.8641
Observations	166	165

**Table 5:** Goodness-of-fit testing for two models.

Independent Variables	Dependent Variables	
	Publications—Country Success	Times Cited—Country Success
	Model 1 (CSPN)	Model 2 (CSPC)
GDP per capita	0.7725 *** (1.2062)	0.6368 *** (7.1524)
GDP per capita in PPP	0.6975 *** (8.4298)	0.6467 *** (7.3418)
Ease of doing business ranking	−0.4821 *** (−4.7652)	−0.4390 *** (−4.2317)
Corruption perceptions index	0.7624 *** (1.5319)	0.6341 *** (7.1014)
Human development index	0.6717 *** (7.8530)	0.5347 *** (5.4799)
Global gender gap	0.4797 *** (4.7348)	0.3354 *** (3.0834)
Happiness index	0.7037 *** (8.5774)	0.5315 *** (5.4340)
Environmental performance index	0.6939 *** (8.3444)	0.5166 *** (5.2256)
Freedom and control	−0.5808 *** (−6.1782)	−0.3832 *** (−3.5932)
Economic freedom	0.6535 *** (7.4765)	0.5801 *** (6.1681)
Democracy Index	0.6227 *** (6.8912)	0.4429 *** (4.2777)
Unemployment rate	−0.1860 (−1.6398)	−0.1642 (−1.4412)
Healthy life expectancy	0.6312 *** (7.0471)	0.5194 *** (5.2635)
Fragile state index	−0.7229 *** (−9.0606)	−0.5405 *** (−5.5634)
Economic decline index	−0.6358 *** (−7.1339)	−0.5597 *** (−5.8487)

Standardized beta coefficients: *** significant at α = *p* < 0.001.

**Table 6:** Descriptive statistics for two models.

Descriptive Statistics	Descriptive Statistics of 2 Models	
	Publications—Country Success	Times Cited—Country Success
	Model 1 (CSPN)	Model 2 (CSPC)
Pearson’s correlation coefficient (|r|)	0.6272	0.5142
Coefficient of determination (R^2^)	0.6943	0.5114
Adjusted R^2^	0.6191	0.3912
Standard deviation	0.1557	1.3693
*p* values (probability level)	0.0000	0.0000
F	9.2356	4.2570

**Table 7:** Standardized beta coefficient values of the dependent variables.

Independent Variables		Standardized Beta Coefficient Values of the Dependent Variables	
		Publications—Country Success	Times Cited—Country Success
		Model 1 (CSPN)	Model 2 (CSPC)
1	GDP per capita	0.7735 **	−0.0853
2	GDP per capita in PPP	−0.5123 *	0.5304 *
3	Ease of doing business ranking	0.2535	0.1599
4	Corruption perceptions index	0.2392	0.3633
5	Human development index	0.1697	−0.1836
6	Global gender gap	−0.0228	0.0703
7	Happiness index	0.0800	−0.0916
8	Environmental performance index	−0.0601 **/	0.1819
9	Freedom and control	−0.0299	0.0846
10	Economic freedom	0.4558	0.3239
11	Democracy Index	−0.1524	0.0577
12	Unemployment rate	0.0353	0.0552
13	Healthy life expectancy	0.0047	0.0696
14	Fragile state index	−0.0008	0.0246
15	Economic decline index	0.0147	−0.0301

Standardized beta coefficients: * significant at—*p* < 0.1, ** significant at *p* < 0.01.

**Table 8:** How country success and its factors influence the two indicators.

Publications—Country Success	Times Cited—Country Success
Model 1 (CSPN)	Model 2 (CSPC)
When a country’s success increases by 1%, the indicator improves by	
1.962%	2.101%
The 17 independent variables explain the dependent variable under analysis by	
89.5%	54.0%

**Table 9:** Benefits and limitations of biometric technologies.

Tool	Benefits	Limitations
Electroencephalography (EEG)	Can be used to measure rapid changes in neural activity by the millisecond [588] Minimally invasive and/or commercial research packages are available [588] Participants can move around and benefit from enriched/social environments [588] Uses portable instruments and natural environments; there is long tradition of well-controlled experiments; measurement processes requiring several hours are possible in practice [589]	It is difficult to pinpoint neural signals from particular brain areas (poor spatial resolution) [588] Measurements from structures deep within the brain (e.g., nucleus accumbens) are not possible [588] Published studies on biometrics based on this signal have used high-cost medical equipment [590] Subjects have reported discomfort since it is necessary to apply scalp neck gel to improve conduction between electrodes [590]
Functional magnetic resonance imaging (fMRI)	Has the ability to observe activity in small structures [588] Differentiates signal from neighboring areas [588] Measurements of the whole brain are possible [588]	Physically restrictive; participants lie on their back in the scanner and cannot move around [588] Expensive, and equipment is in high demand [588] Equipment cannot be removed from the laboratory; the sequence of the activities is difficult to monitor [589]
MEG (magnetoencephalography)	Some MEG study protocols are quite well suited for design studies; there is a long tradition of well-controlled experiments based on EEG; optimal space-time-resolution [589]	Equipment cannot be removed from the laboratory; the location of existing brain activity is relatively difficult to determine [589]
Electrocardiogram (ECG)	Highly reliable source providing precise features of the electrical and physiological activity taking place with an individual; high performance has been noted in prior research on this signal [591]; it can easily be fused with other signals [592]	One of the great difficulties listed in the literature is a lack of user acceptance, as its implementation at the physical level makes it fairly uncomfortable [593]; body posture can also affect cardiac signals [594]
MRI (magnetic resonance imaging) [589]	Good for studies comparing groups of people	Equipment cannot be removed from the laboratory
PET (positron emission tomography) [589]	Good for comparing groups of people or natural tasks	Radioactive tracer is injected into participants; equipment cannot be removed from the laboratory
Eye tracking [588]	Offers strong nuanced data on visual attention and gaze pathways, and can be integrated with pupillometry	Does not measure inferences, the valence of the response, thoughts, or emotions
Iris [595]	Unique data; input is stable throughout lifetime; non-intrusive	Large data template; images are frequently improperly focused; single-source; high cost
NIRS (near-infrared spectroscopy) [589]	Uses portable instruments and natural environments; some NIRS study protocols are well suited for design studies; measurement processes requiring several hours are possible in practice	Difficulties in determining the location of brain activity; few groups are using NIRS for cognitive studies as yet
Transcranial magnetic stimulation (TMS/tDCS) [588]	Can be used to show causality	Limited to investigating the function of brain surface areas Can generally only lessen (TMS/tDCS) or increase (tDCS) neural activity in a general sense; cannot test for specific levels of activity or influence specific circuits
Forehead electrooculogram (EOG)	These signals are low cost, and are not invasive [596]	Electrodes used for the acquisition of the signals can present instability to eye flicker [597]; signals are highly affected by noises in the immediate vicinity [596]
Skin conductance response (SCR), heart rate, pupil dilation [588]	Simple; well validated Unobtrusive equipment; allows for more natural interactions with the environment	Cannot distinguish between positive and negative arousal
Lips [598]	Easy acquisition and lip characteristics; it is possible to extract the outline even if the person has a beard or a moustache	An image of the lips cannot be acquired when they are moving
Facial electromyography (fEMG), facial affective coding [599]	This is a precise and sensitive method for measuring emotional expression Unlike self-reports, fEMG does not depend on language and does not require cognitive effort or memory Yields large amounts of data and is continuous and scalable (hence more credible) Dynamic tracking of emotional (potentially unconscious) responses to ongoing stimuli/information Can measure facial muscle activities for the sake of balancing weakly evocative emotional stimuli Less intrusive than other physiological measures such as fMRI and EEG Automatic facial encoding software/algorithms are available	The technique is intrusive and may alter natural expression The number of muscles that can be triggered is limited by how many electrodes can be attached to the face Requires electrodes to be directly attached to the face (in a lab) Certain medicines that act on the nervous system, such as muscle relaxants and anticholinergics, can impact the final electromyography (EMG) result
Gait	Convenient and non-intrusive (2D); subjects can be evaluated covertly, without their knowledge [595]	During the assessment stage, light affects the results; clothing may affect detection [46] Data may alter throughout a lifetime (injuries, training, footwear); specialist personnel required for data processing; large data template [595]
Body motion [595]	Unique and various sources of data, small template size	Time consuming; subject must cooperate with reader; specialist personnel required for data processing

**Table 10:** Comparison of biometric technologies by seven characteristics (traits).

	Universality	Uniqueness or Distinctiveness	Permanence	Collectability	Performance	Acceptability	Circumvention
Iris/pupil	High [141,602,603,604,605,606]	High [141,602,603,604,605,606]	High [141,602,603,604,605,606]	Medium [141,602,603,604,605,606]	High [141,602,603,604,605,606]	Low [141,602,604,605,606] Medium [603]	High [141,602] Low [603,604,605,606]
Face	High [141,602,603,604,605,606]	Low [141,602,604,605,606] Medium [603]	Medium [141,602,603,604,605,606]	High [141,602,603,604,605,606]	Low [141,602,603,604,605,606]	High [141,602,603,604,605,606]	Low [141,602] High [603,604,605,606]
Odor	High [602,603,604,605,606]	High [602,603,604,605,606]	High [602,603,604,605,606]	Low [602,603,604,605,606]	High [602,603] Low [604,605,606]	Low [602] Medium [603,604,605,606]	Low [602,603,604,605,606]
Keystroke dynamics and mouse movements, Mouse Tracking	Low [141,602,604,605,606]	Low [141,602,604,605,606]	Low [141,602,604,605,606]	Medium [141,602,604,605,606]	Low [141,602,604,605,606]	Medium [141,602,604,605,606]	Medium [141,602,604,605,606]
Skin temperature -thermogram	High [141,604,605,606]	High [141,604,605,606]	Low [141,604,605,606]	High [141,604,605,606]	Medium [141,604,605,606]	High [141,604,605,606]	High [141] Low [604,605,606]
Voice/Speech/Voice Pitch Analysis (VPA)	Medium [141,602,604,605,606]	Low [141,602,604,605,606]	Low [141,602,604,605,606]	Medium [141,602,604,605,606]	Low [141,602,604,605,606]	High [141,602,604,605,606]	Low [141,602] High [604,605,606]
Signature	Low [141,602,603,604,605,606]	Low [141,602,603,604,605,606]	Low [141,602,603,604,605,606]	High [141,602,603,604,605,606]	Low [141,602,604,605,606] Medium [606]	High [141,602,603,604,605,606]	Low [141,602] High [603,604,605,606]
Gait	Medium [602,604,605,606] High [603]	Low [141,604,605,606] Medium [603]	Low [141,604,605,606] Medium [603]	High [602,603,604,605,606]	Low [602,603,604,605,606]	High [602,604,605,606] Medium [603]	Medium [602,603,604,605,606]

**Table 11:** Comparison of biometric technologies by various attributes.

	Easy of Use	Error Incidence	Accuracy	User Acceptance	Long Term Stability	Cost	Size of Template	Security	Socially Introduced	Social Acceptability	Popularity	Speed
Eye Tracking (ET)			0.5°–1° [607]			Low-High [608]						
Iris/pupil	Medium [602,603,609]	Lighting [602,609] Lighting, glasses [603]	Very High [602,609] High [320,603,610,611]	Medium [602,609]	High [602,609,610] Medium [320,603]	High [320,603,611]	Small [320]	Medium [320] High [603] Very high [609]	1995 [603]	Medium-Low [610,611]	Medium [603]	Medium [603]
Face	Medium [602,609] High [603]	Lighting, age glasses, hair [602,603,609]	High [602,609] Low [320,602] Medium-Low [610,611]	Medium [602,609]	Medium [602,609] Low [320,602]	High [320] Medium [602,610,611]	Large [320]	Low [320] Medium [602,610,611]	2000 [603]	High [610,611]	High [603]	Medium [603]
Keystroke dynamics and mouse movements, Mouse Tracking	Low [602]	Device, weather [602]	Low [602]		Low [602]	Medium [602]		Low [602]	2005 [603]		Low [603]	Medium [603]
Voice/Speech/Voice Pitch Analysis (VPA)	High [602,603,609]	Noise, colds [602,603,609]	High [602,609] Low [320,603] Medium [610,611]	High [602,609]	Medium [602,603,609] Low [320]	Medium [320,610,611] Low [603]	Small [320]	Low [320] High [603] Medium [609]	1998 [603]	High [610,611]	High [603]	High [603]
Signature	High [602,603,609]	Changing signature [602,603,609]	High [602,609] Medium [320,603] Low [610,611]	High [602] Very High [609]	Medium [602,609] Low [320,603]	Low [320] Medium [603,610,611]	Medium [320]	Low [320] High [603] Medium [609]	1970 [603]	High [610,611]	High [603]	High [603]
Gait			Medium [610]			Medium [610]				Low [610]		
Lip Movement			Medium [603]		Medium [603]	Medium [603]	Small [603]	High [603]				
Gesture			Low [612]									

**Table 12:** Comparison of performance metrics for biometric technologies by various authors.

	FAR	FRR	CER	FTE
Iris/pupil	0.94% [603] 0.0001–0.94 [613] 2.4649% [614]	0.99% [603] 0.99–0.91 [613] 2.4614% [614]	0.01% [603]	0.50% [603]
Face	1% [603] 16% [614]	10% [603] 16% [614]		3.1% [615]
Keystroke dynamics and mouse movements, Mouse Tracking	7% [603] 0.01% [614]	0.10% [603] 4% [614]	1.80% [603]	
Voice/Speech/Voice Pitch Analysis (VPA)	2% [603,613] 7% [614]	10% [603,613] 7% [614]	6% [603]	0.5% [615]

## Data Availability

All extracted data are included in the manuscript.

## Abbreviations

AFFECT	arousal, valence, affective attitudes, emotional and physiological states
AI	Artificial Intelligence
AISs	artificial intelligence subsystems
AON	action observation network
BAEPs	brainstem evoked potentials
BCI	brain–computer interface
BIM4Ren	Building Information Modelling based tools and technologies toward fast and efficient RENovation of residential buildings
BNCI	brain/neuronal computer interaction
BR	binary relevance
BTL	below the line
CNCP	model Collective Neuromarketing Consumer Persuasion Model
CNN	convolutional neural network
DEAP	Dataset for Emotions Analysis using Physiological signals
DTI	diffusion tensor imaging
DWI	diffusion-weighted imaging
EARs	emotion association rules
ECG	electrocardiography
EDA	electrodermal activity
EEG	electroencephalography
EMG	electromyography
EMSs	engagement marketing subsystems
EOG	electrooculogram
ERP	event-related potential
ESSs	emotional salient segments
ET	eye tracking
FA	fractional anisotropy
FC	facial action coding
FDG	fluoro-D-glucose
FDG-PET/fMRI	simultaneous [18 F]-fluorodeoxyglucose positron emission tomography and functional magnetic resonance imaging
fEMG	facial electromyography
fMRI	functional magnetic resonance imaging
fNIRS	functional near-infrared spectroscopy
fPET	functional positron emission tomography
GMM	gaussian mixture models
GSR	galvanometer or galvanic skin response
HMI	human–machine interactions
HMM	hidden Markov model
HR	heart rate
HVAC	heating, ventilation, and air conditioning
ICCs	intra-class correlation coefficients
IoT	Internet of Things
IRT	implicit reaction time
IS	information systems
iTBS	intermittent theta burst transcranial magnetic stimulation
K-NN	K-nearest neighbor
LP	label powerset
LSTM	long short-term memory
MDS	multidimensional scaling
MEG	magnetoencephalography
MLP	multi-layer perceptron
MRI	magnetic resonance imaging
MT	mouse tracking
N5PSC	neuromarketing, neuroeconomics, neuromanagement, neuro-information systems, neuro-industrial engineering, products, services, call centers
NEV	net emotional value
NIRS	near infrared spectroscopy
NLP	natural language processing
NT	neurotransmitter
PET	positron emission tomography
PPG	photoplethysmogram
PSD	power spectral density
RAKEL	random k-label sets
RF	random forest
ROCK	Regeneration and Optimization of Cultural heritage in creative and Knowledge cities
RRA	respiratory rate assessment
RT	reaction times
rTMS	transcranial magnetic stimulation
SC	skin conductance
SD	tests SDS denaturation test
SEEVal	the service encounter emotional value
SST	steady-state topography
SVM	support vector machine
tDCS	transcranial direct-current stimulation
TMS	transcranial magnetic stimulation
UIT-VSMEC	standard Vietnamese social media emotion corpus
VAAQ	virtual agent’s acceptance questionnaire
VPA	voice pitch analysis
VR	virtual reality
